# Supplementary material for: Meta-analysis of public RNA-sequencing data of drought and salt stresses in different phenotypes of resistant and susceptible Oryza sativa cultivars
Source: Quant Plant Biol. 2025 Sep 5;6:e27. doi: 10.1017/qpb.2025.10020 (PMC12451249; doi:10.1017/qpb.2025.10020)
Supplement: Shintani and Bono supplementary material [file S2632882825100209sup001.zip › Supplementary_FigureS2.pdf]

(a)

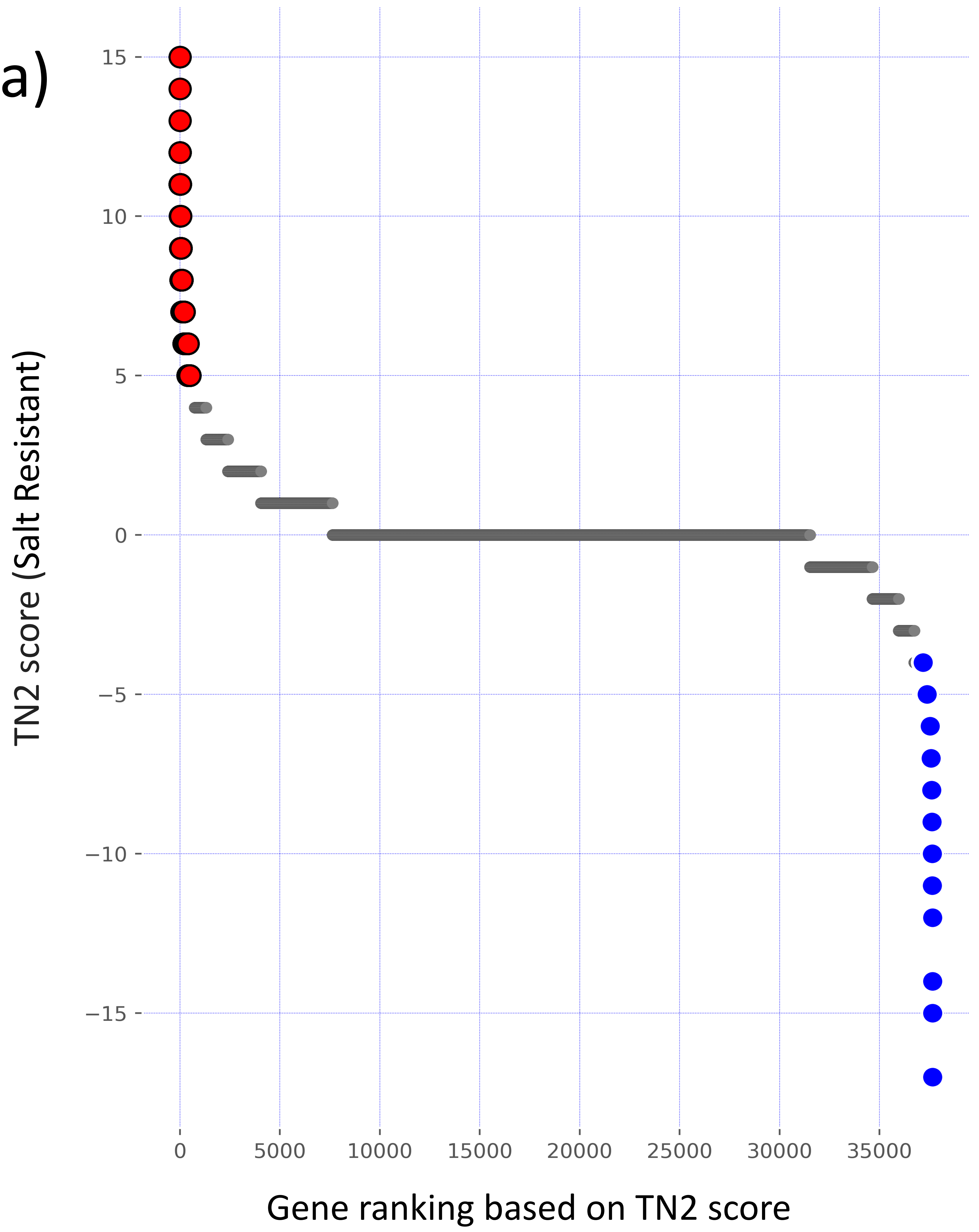

15
10
5
0
−5
−10
−15
TN2 score (Salt Resistant)
0
5000
10000
15000
20000
25000
30000
35000
Gene ranking based on TN2 score

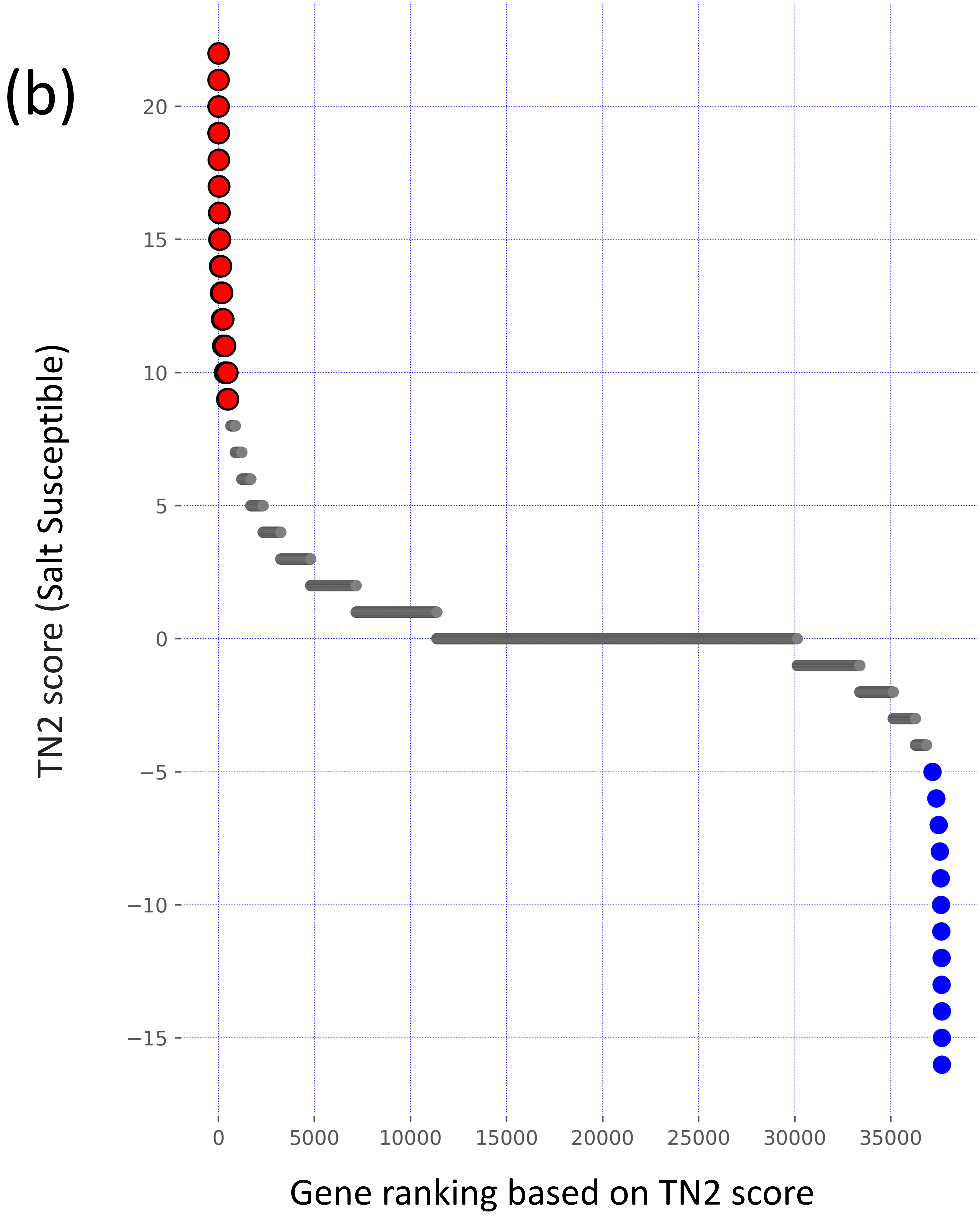
(b)
TN2 score (Salt Susceptible)
Gene ranking based on TN2 score
20
15
10
5
0
−5
−10
−15
0
5000
10000
15000
20000
25000
30000
35000

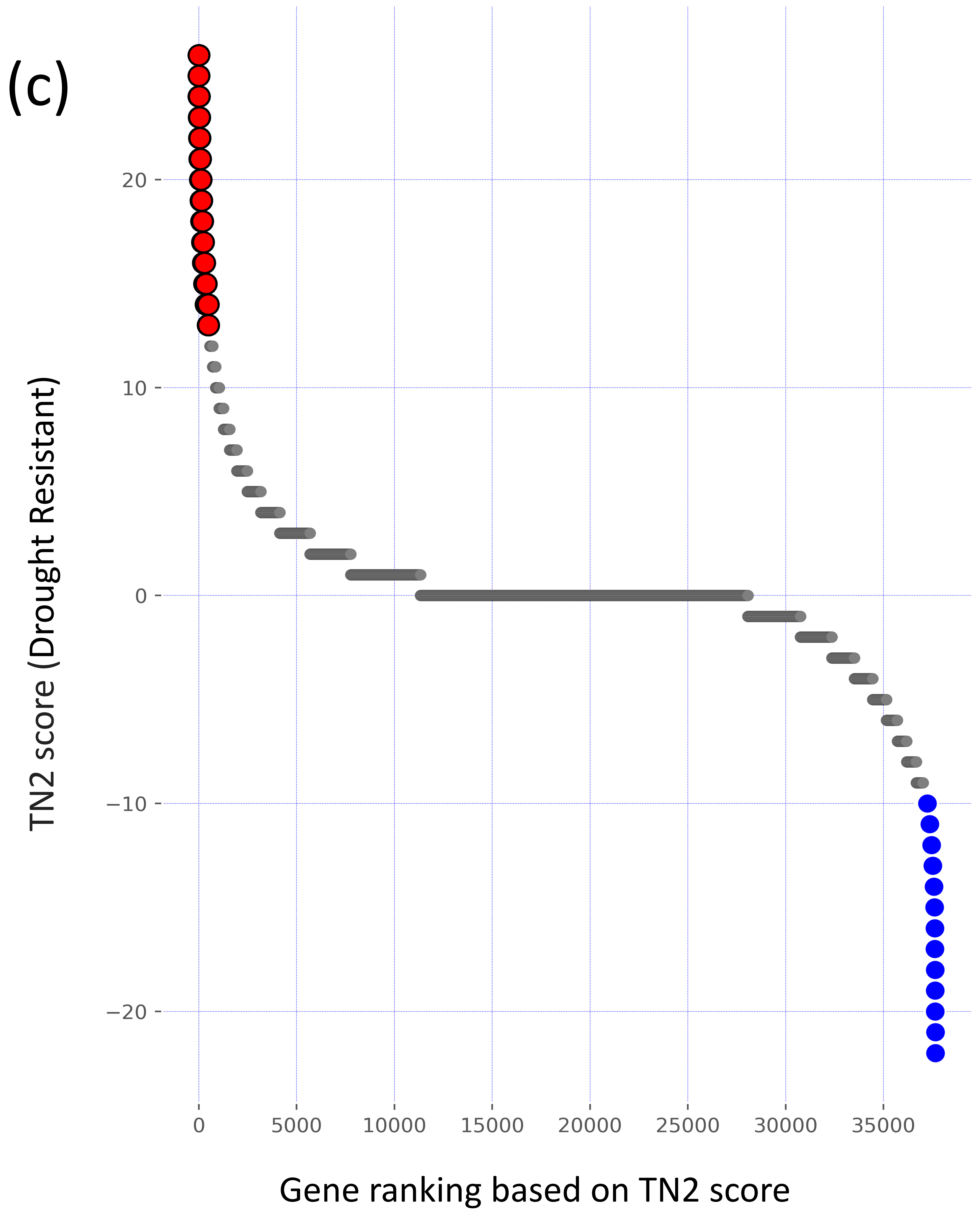
(c)
TN2 score (Drought Resistant)
20
10
0
−10
−20
0
5000
10000
15000
20000
25000
30000
35000
Gene ranking based on TN2 score

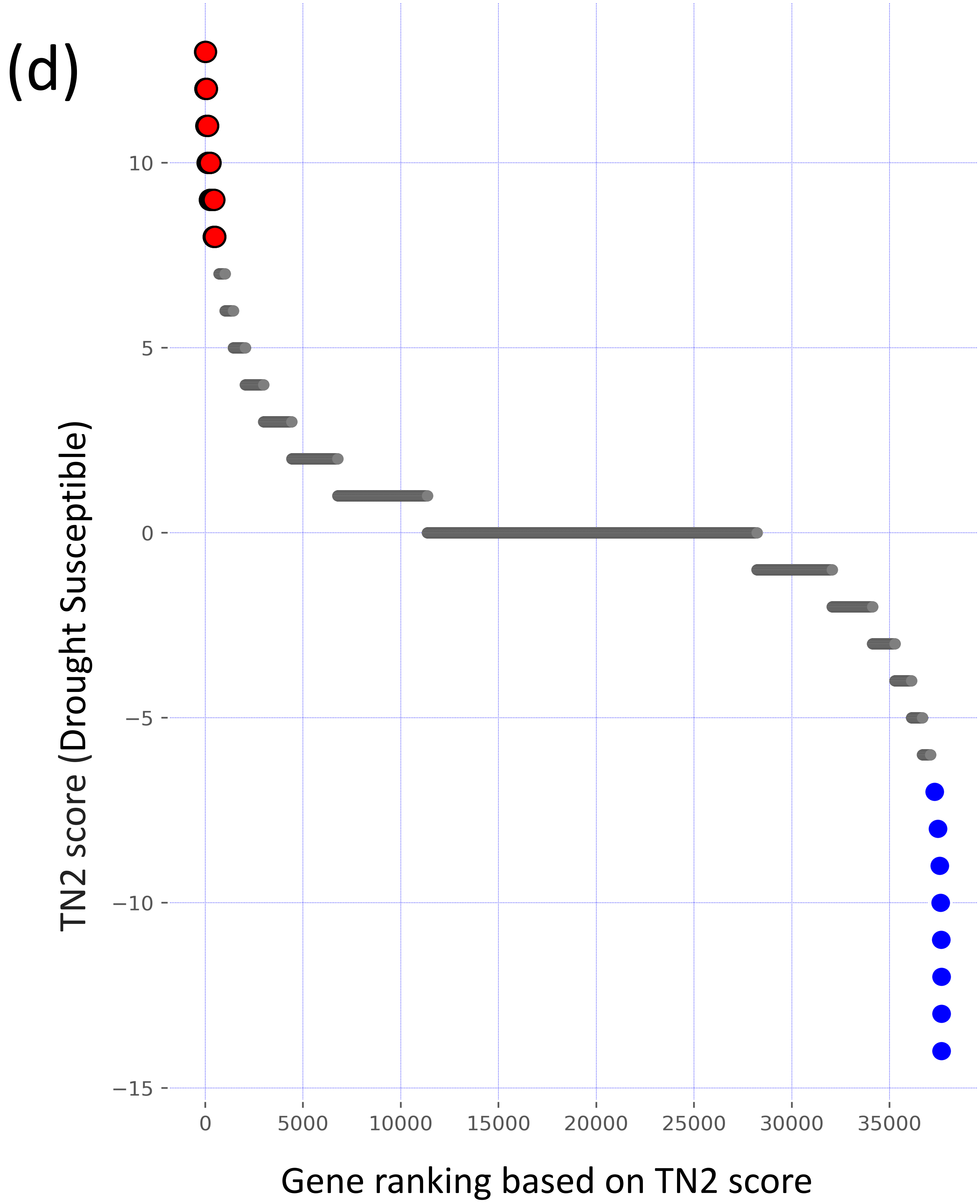
(d)
TN2 score (Drought Susceptible)
10
5
0
−5
−10
−15
0
5000
10000
15000
20000
25000
30000
35000
Gene ranking based on TN2 score
