# Supplementary material for: Meta-analysis of public RNA-sequencing data of drought and salt stresses in different phenotypes of resistant and susceptible Oryza sativa cultivars
Source: Quant Plant Biol. 2025 Sep 5;6:e27. doi: 10.1017/qpb.2025.10020 (PMC12451249; doi:10.1017/qpb.2025.10020)
Supplement: Shintani and Bono supplementary material [file S2632882825100209sup001.zip › Supplementary_FigureS4.pdf]

(a)

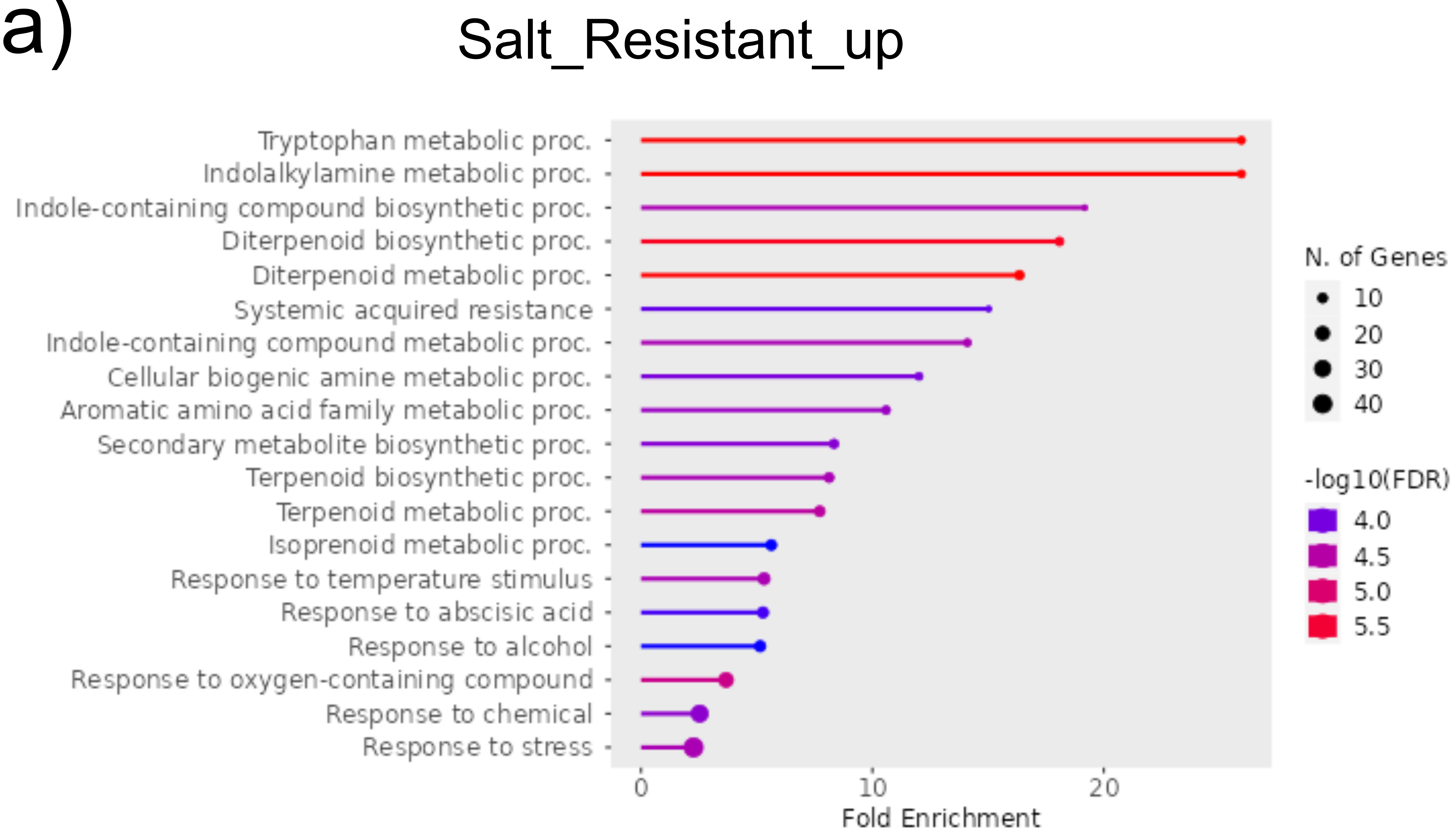
Salt_Resistant_up
Tryptophan metabolic proc.
Indolalkylamine metabolic proc.
Indole-containing compound biosynthetic proc.
Diterpenoid biosynthetic proc.
Diterpenoid metabolic proc.
Systemic acquired resistance
Indole-containing compound metabolic proc.
Cellular biogenic amine metabolic proc.
Aromatic amino acid family metabolic proc.
Secondary metabolite biosynthetic proc.
Terpenoid biosynthetic proc.
Terpenoid metabolic proc.
Isoprenoid metabolic proc.
Response to temperature stimulus
Response to abscisic acid
Response to alcohol
Response to oxygen-containing compound
Response to chemical
Response to stress
0
10
20
Fold Enrichment
N. of Genes
10
20
30
40
-log10(FDR)
4.0
4.5
5.0
5.5

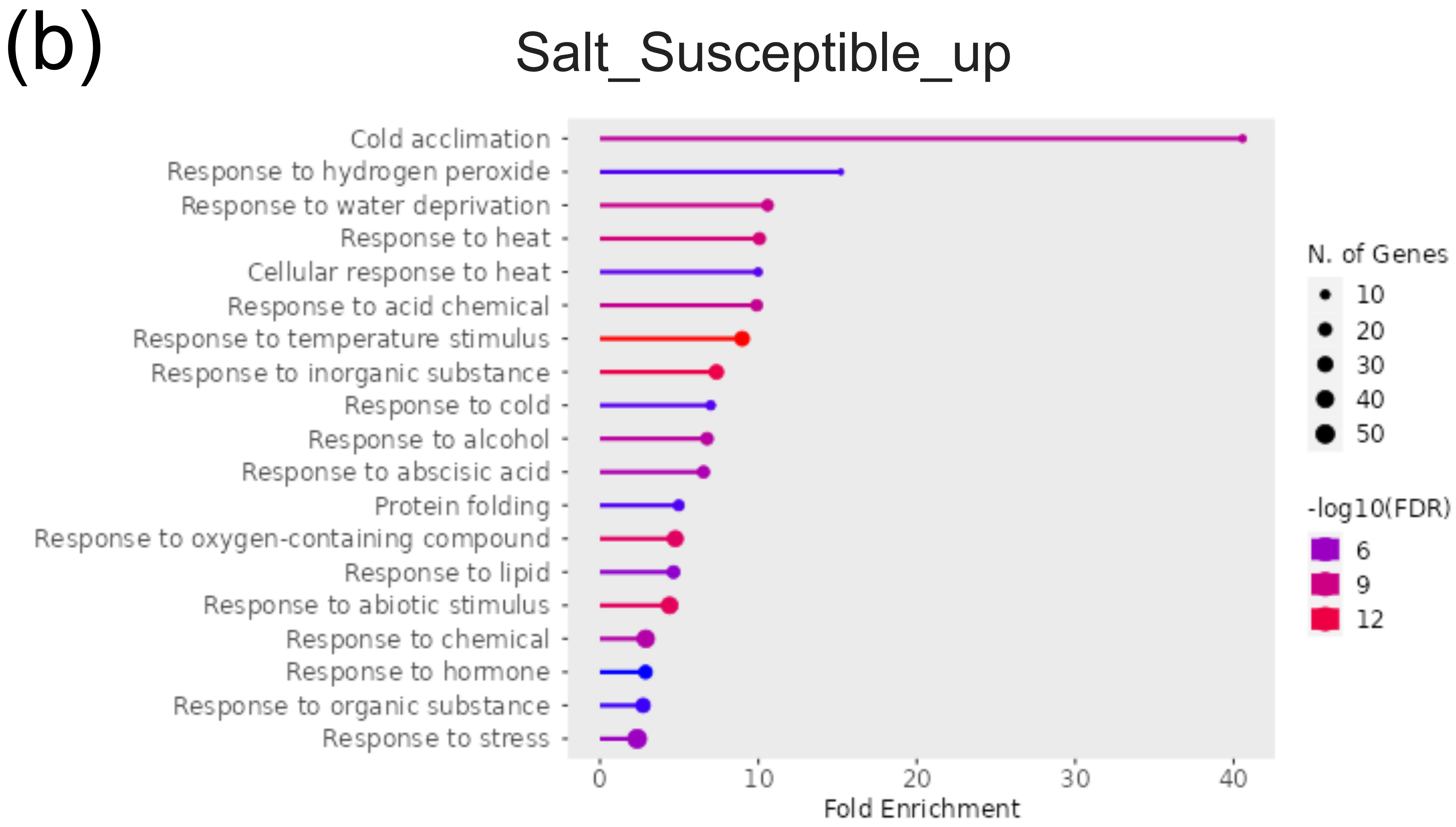
(b)
Salt_Susceptible_up
Cold acclimation
Response to hydrogen peroxide
Response to water deprivation
Response to heat
Cellular response to heat
Response to acid chemical
Response to temperature stimulus
Response to inorganic substance
Response to cold
Response to alcohol
Response to abscisic acid
Protein folding
Response to oxygen-containing compound
Response to lipid
Response to abiotic stimulus
Response to chemical
Response to hormone
Response to organic substance
Response to stress
0
10
20
30
40
Fold Enrichment
N. of Genes
10
20
30
40
50
-log10(FDR)
6
9
12

(c)

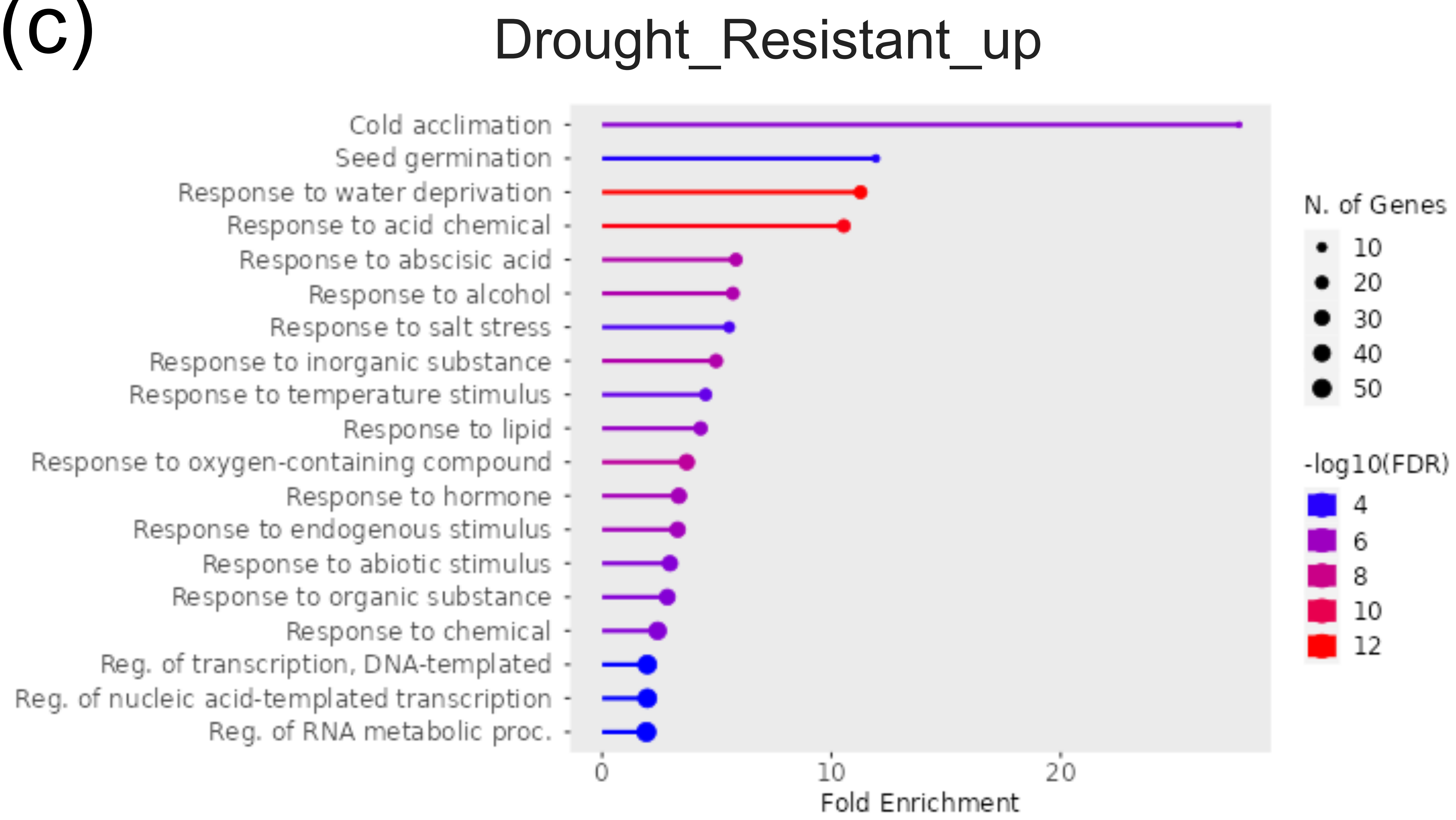
Drought_Resistant_up
Cold acclimation
Seed germination
Response to water deprivation
Response to acid chemical
Response to abscisic acid
Response to alcohol
Response to salt stress
Response to inorganic substance
Response to temperature stimulus
Response to lipid
Response to oxygen-containing compound
Response to hormone
Response to endogenous stimulus
Response to abiotic stimulus
Response to organic substance
Response to chemical
Reg. of transcription, DNA-templated
Reg. of nucleic acid-templated transcription
Reg. of RNA metabolic proc.
0
10
20
Fold Enrichment
N. of Genes
10
20
30
40
50
-log10(FDR)
4
6
8
10
12

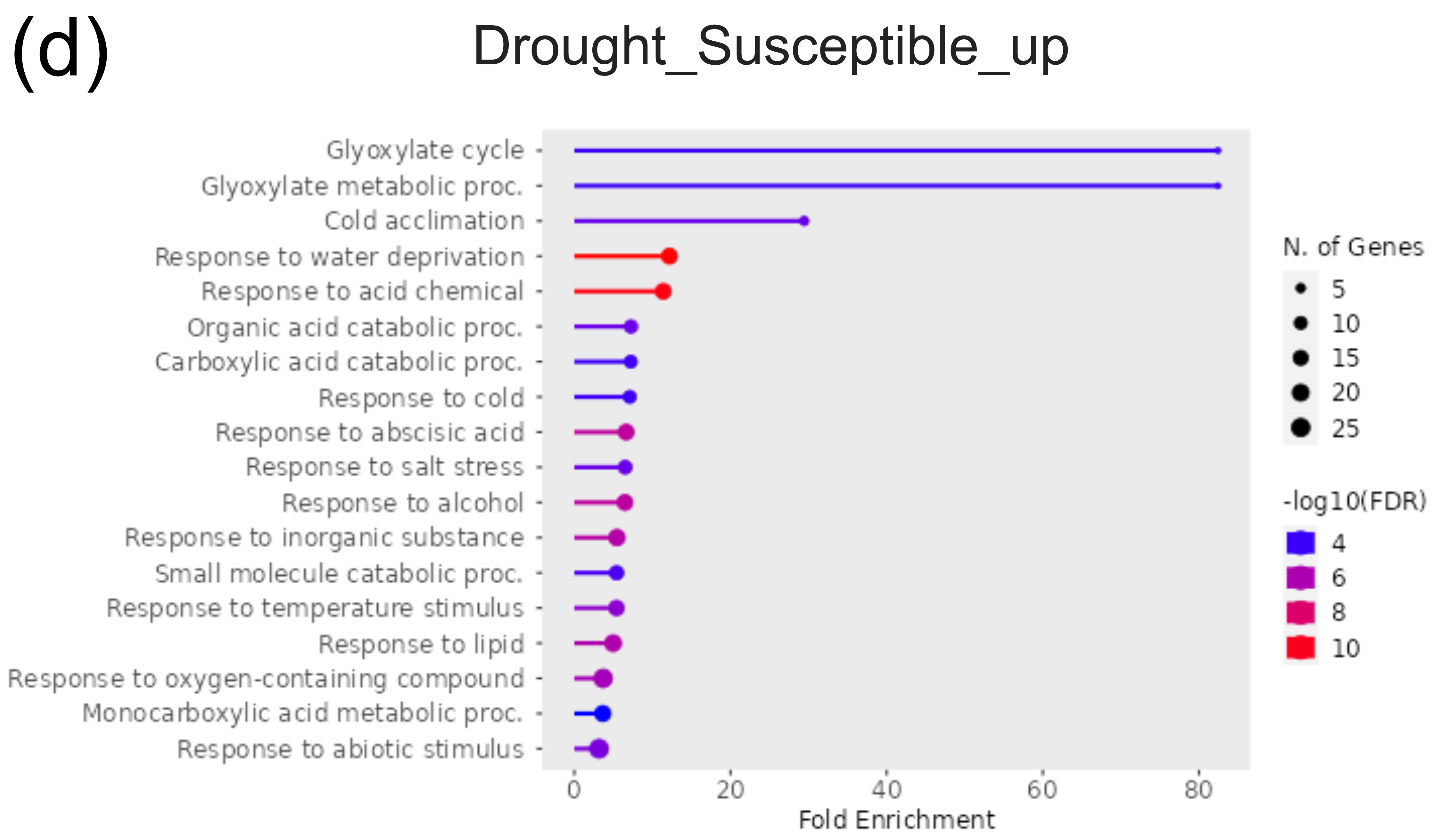

(d)
Drought_Susceptible_up
Glyoxylate cycle
Glyoxylate metabolic proc.
Cold acclimation
Response to water deprivation
Response to acid chemical
Organic acid catabolic proc.
Carboxylic acid catabolic proc.
Response to cold
Response to abscisic acid
Response to salt stress
Response to alcohol
Response to inorganic substance
Small molecule catabolic proc.
Response to temperature stimulus
Response to lipid
Response to oxygen-containing compound
Monocarboxylic acid metabolic proc.
Response to abiotic stimulus
0
20
40
60
80
Fold Enrichment
N. of Genes
5
10
15
20
25
-log10(FDR)
4
6
8
10

(e)

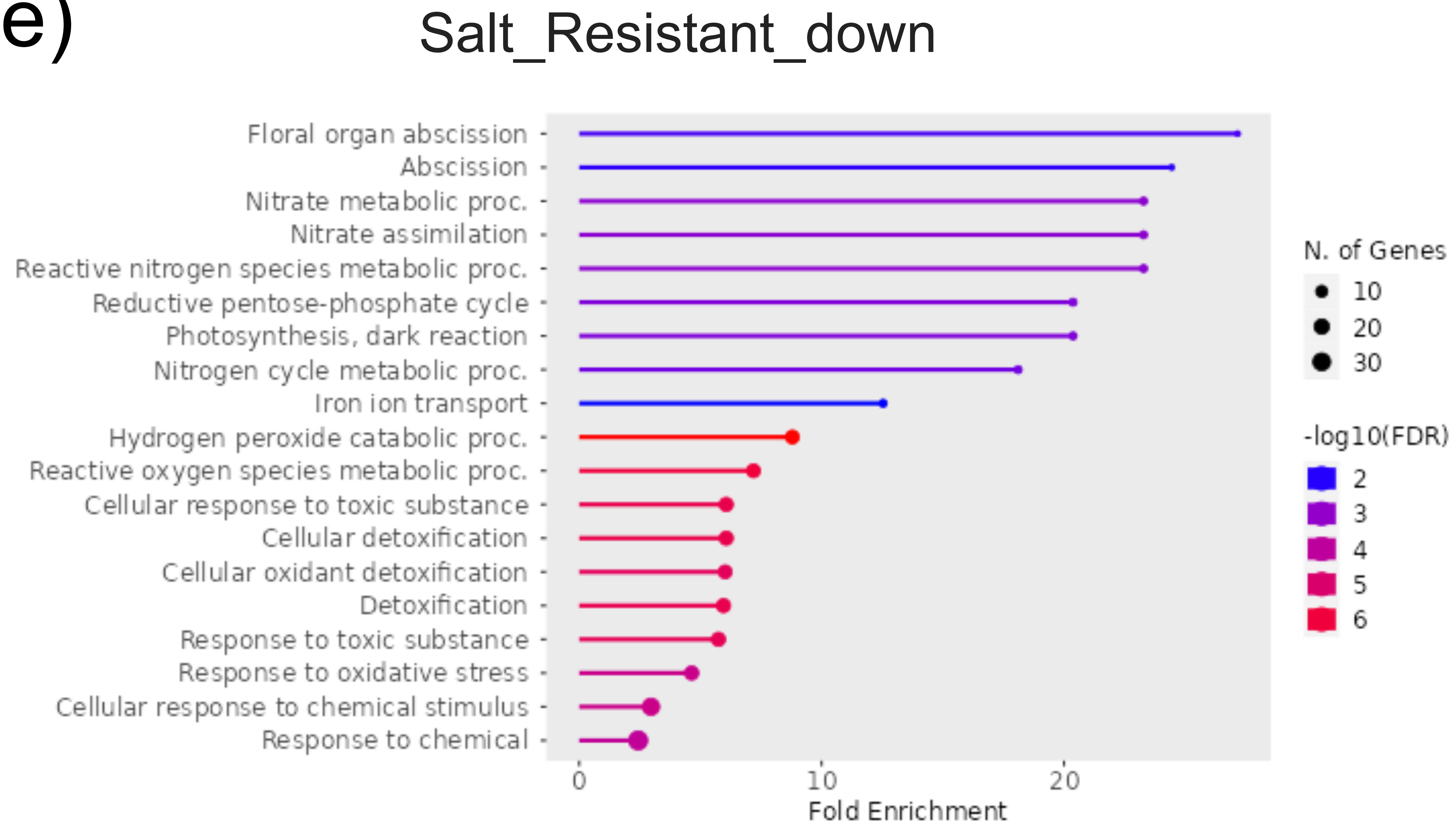

Salt_Resistant_down
Floral organ abscission
Abscission
Nitrate metabolic proc.
Nitrate assimilation
Reactive nitrogen species metabolic proc.
Reductive pentose-phosphate cycle
Photosynthesis, dark reaction
Nitrogen cycle metabolic proc.
Iron ion transport
Hydrogen peroxide catabolic proc.
Reactive oxygen species metabolic proc.
Cellular response to toxic substance
Cellular detoxification
Cellular oxidant detoxification
Detoxification
Response to toxic substance
Response to oxidative stress
Cellular response to chemical stimulus
Response to chemical
0
10
20
Fold Enrichment
N. of Genes
10
20
30
-log10(FDR)
2
3
4
5
6

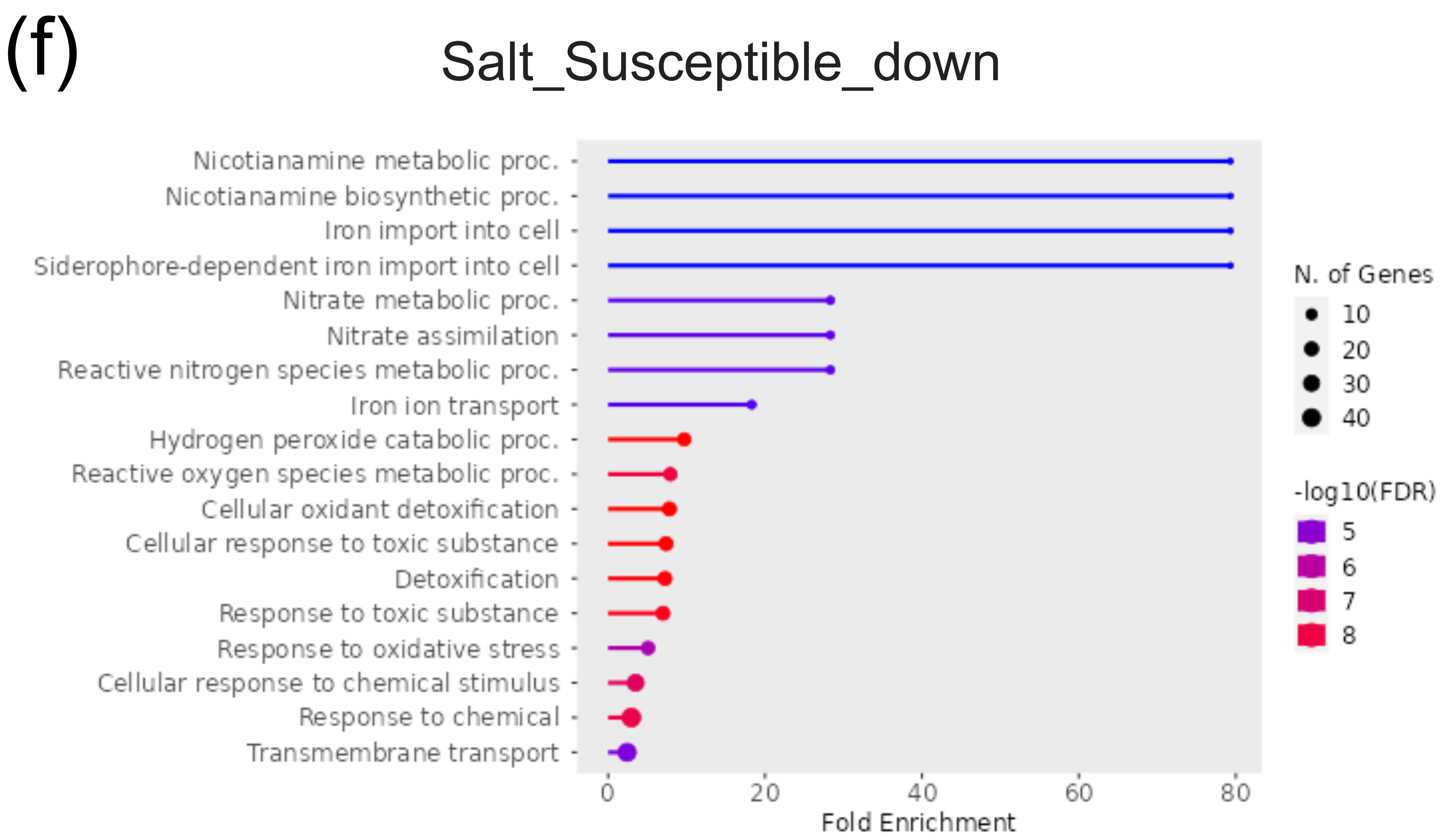
(f)
Salt_Susceptible_down
Nicotianamine metabolic proc.
Nicotianamine biosynthetic proc.
Iron import into cell
Siderophore-dependent iron import into cell
Nitrate metabolic proc.
Nitrate assimilation
Reactive nitrogen species metabolic proc.
Iron ion transport
Hydrogen peroxide catabolic proc.
Reactive oxygen species metabolic proc.
Cellular oxidant detoxification
Cellular response to toxic substance
Detoxification
Response to toxic substance
Response to oxidative stress
Cellular response to chemical stimulus
Response to chemical
Transmembrane transport
0
20
40
60
80
Fold Enrichment
N. of Genes
10
20
30
40
-log10(FDR)
5
6
7
8

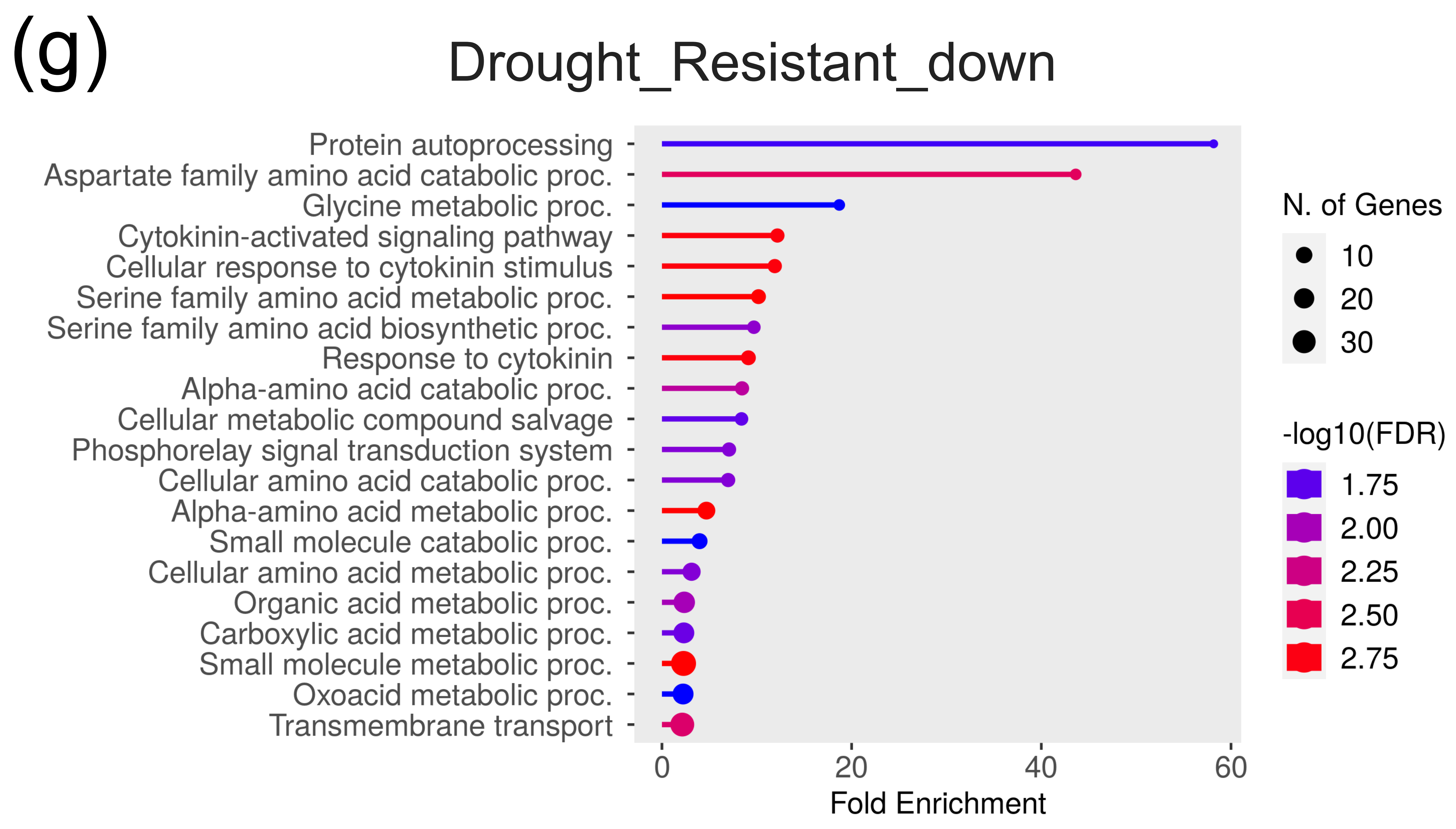
(g)
Drought_Resistant_down
Protein autoprocessing
Aspartate family amino acid catabolic proc.
Glycine metabolic proc.
Cytokinin-activated signaling pathway
Cellular response to cytokinin stimulus
Serine family amino acid metabolic proc.
Serine family amino acid biosynthetic proc.
Response to cytokinin
Alpha-amino acid catabolic proc.
Cellular metabolic compound salvage
Phosphorelay signal transduction system
Cellular amino acid catabolic proc.
Alpha-amino acid metabolic proc.
Small molecule catabolic proc.
Cellular amino acid metabolic proc.
Organic acid metabolic proc.
Carboxylic acid metabolic proc.
Small molecule metabolic proc.
Oxoacid metabolic proc.
Transmembrane transport
0
20
40
60
Fold Enrichment
N. of Genes
10
20
30
-log10(FDR)
1.75
2.00
2.25
2.50
2.75

(h)

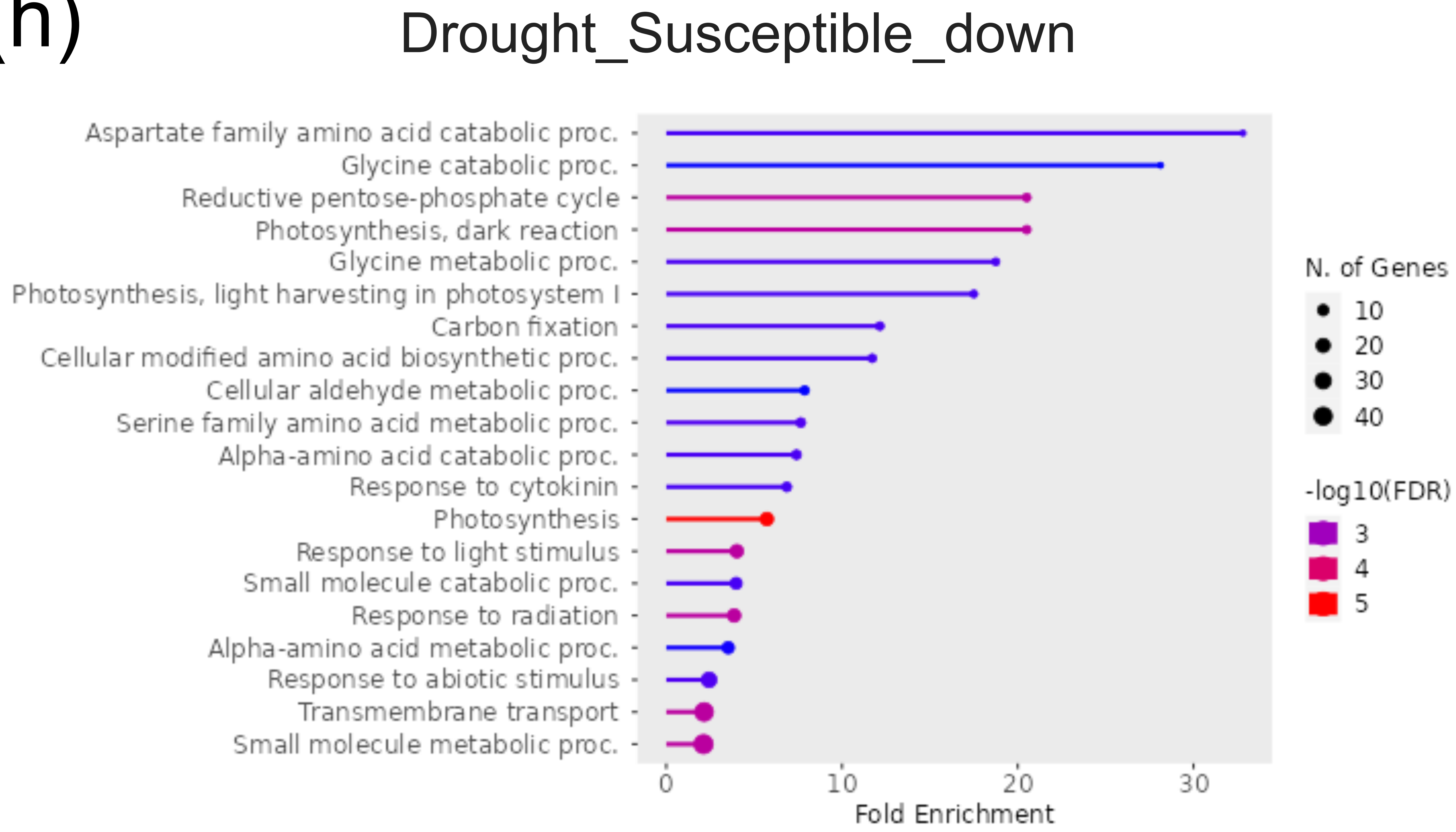
Drought_Susceptible_down
Aspartate family amino acid catabolic proc.
Glycine catabolic proc.
Reductive pentose-phosphate cycle
Photosynthesis, dark reaction
Glycine metabolic proc.
Photosynthesis, light harvesting in photosystem I
Carbon fixation
Cellular modified amino acid biosynthetic proc.
Cellular aldehyde metabolic proc.
Serine family amino acid metabolic proc.
Alpha-amino acid catabolic proc.
Response to cytokinin
Photosynthesis
Response to light stimulus
Small molecule catabolic proc.
Response to radiation
Alpha-amino acid metabolic proc.
Response to abiotic stimulus
Transmembrane transport
Small molecule metabolic proc.
0
10
20
30
Fold Enrichment
N. of Genes
10
20
30
40
-log10(FDR)
3
4
5
