# Supplementary material for: Meta-analysis of public RNA-sequencing data of drought and salt stresses in different phenotypes of resistant and susceptible Oryza sativa cultivars
Source: Quant Plant Biol. 2025 Sep 5;6:e27. doi: 10.1017/qpb.2025.10020 (PMC12451249; doi:10.1017/qpb.2025.10020)
Supplement: Shintani and Bono supplementary material [file S2632882825100209sup001.zip › Supplementary_FigureS5.pdf]

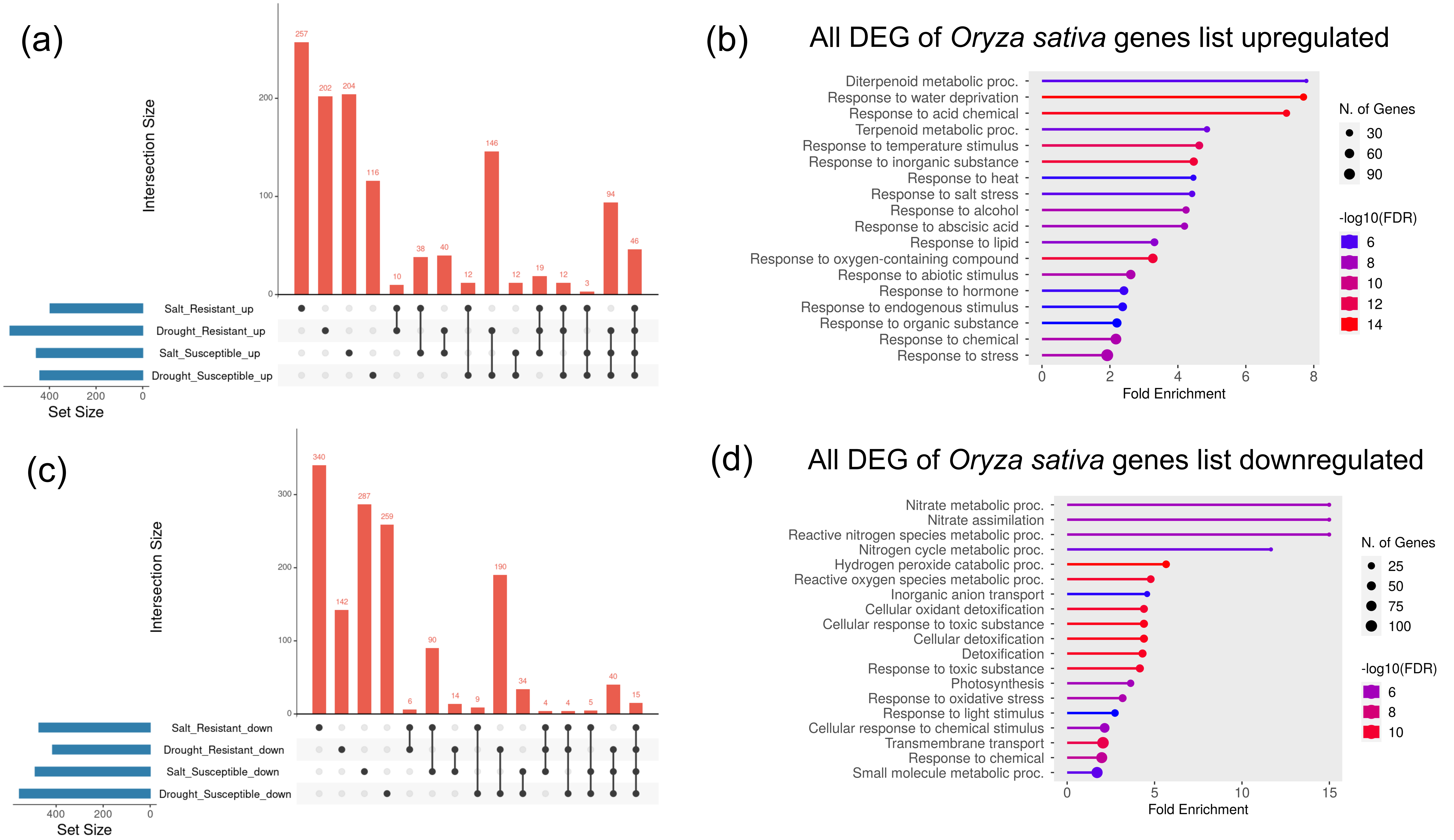

(a)
Intersection Size
257
202
204
116
10
38
40
12
146
12
19
12
3
94
46
Salt_Resistant_up
Drought_Resistant_up
Salt_Susceptible_up
Drought_Susceptible_up
Set Size
(b)
All DEG of *Oryza sativa* genes list upregulated
Diterpenoid metabolic proc.
Response to water deprivation
Response to acid chemical
Terpenoid metabolic proc.
Response to temperature stimulus
Response to inorganic substance
Response to heat
Response to salt stress
Response to alcohol
Response to abscisic acid
Response to lipid
Response to oxygen-containing compound
Response to abiotic stimulus
Response to hormone
Response to endogenous stimulus
Response to organic substance
Response to chemical
Response to stress
Fold Enrichment
N. of Genes
-log10(FDR)
(c)
Intersection Size
340
142
287
259
6
90
14
9
190
34
4
4
5
40
15
Salt_Resistant_down
Drought_Resistant_down
Salt_Susceptible_down
Drought_Susceptible_down
Set Size
(d)
All DEG of *Oryza sativa* genes list downregulated
Nitrate metabolic proc.
Nitrate assimilation
Reactive nitrogen species metabolic proc.
Nitrogen cycle metabolic proc.
Hydrogen peroxide catabolic proc.
Reactive oxygen species metabolic proc.
Inorganic anion transport
Cellular oxidant detoxification
Cellular response to toxic substance
Cellular detoxification
Detoxification
Response to toxic substance
Photosynthesis
Response to oxidative stress
Response to light stimulus
Cellular response to chemical stimulus
Transmembrane transport
Response to chemical
Small molecule metabolic proc.
Fold Enrichment
N. of Genes
-log10(FDR)
