# Supplementary material for: Meta-analysis of public RNA-sequencing data of drought and salt stresses in different phenotypes of resistant and susceptible Oryza sativa cultivars
Source: Quant Plant Biol. 2025 Sep 5;6:e27. doi: 10.1017/qpb.2025.10020 (PMC12451249; doi:10.1017/qpb.2025.10020)
Supplement: Shintani and Bono supplementary material [file S2632882825100209sup001.zip › Supplementary_FigureS6.pdf]

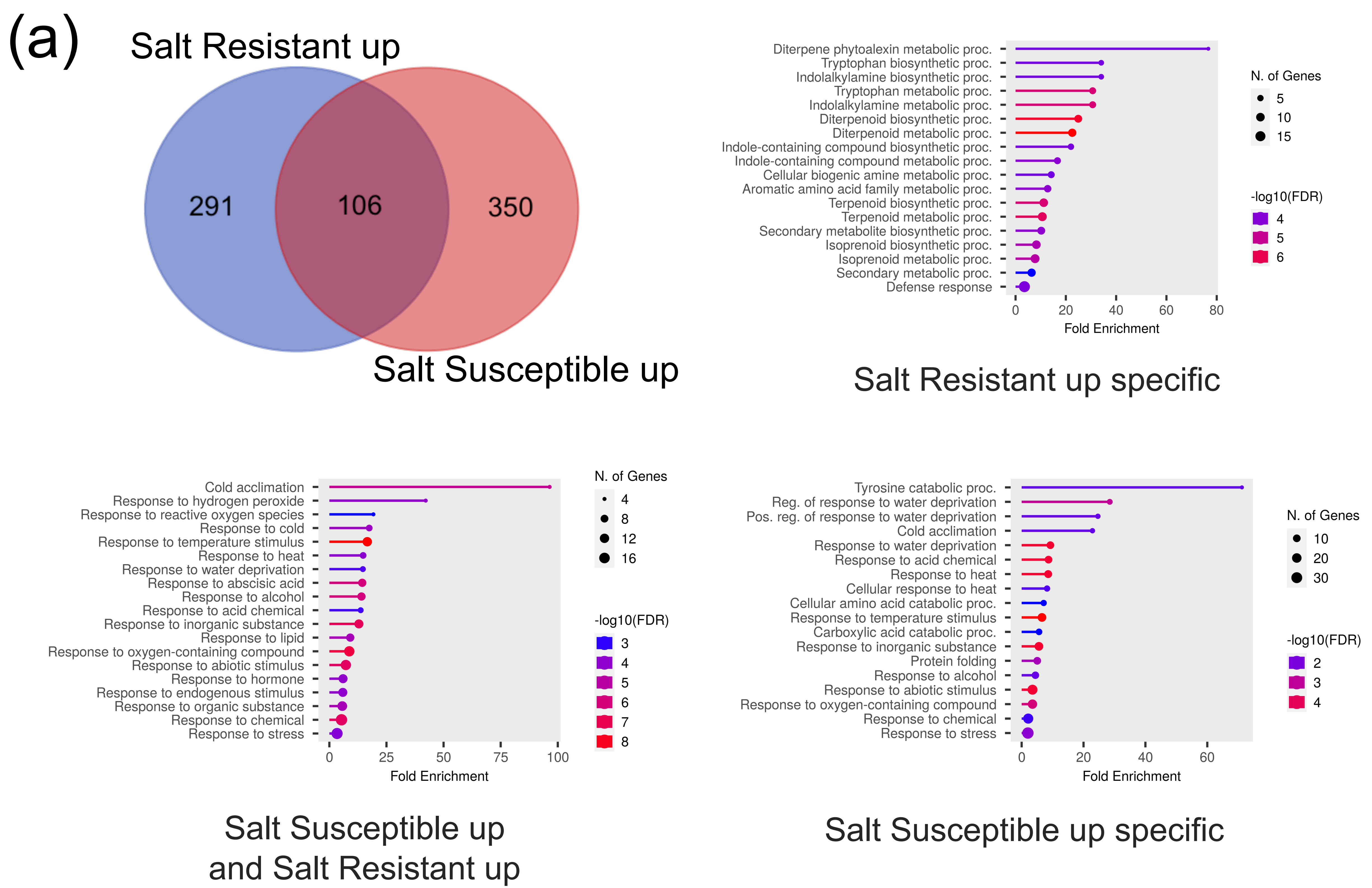
(a)
Salt Resistant up
291
106
350
Salt Susceptible up
Diterpene phytoalexin metabolic proc.
Tryptophan biosynthetic proc.
Indolalkylamine biosynthetic proc.
Tryptophan metabolic proc.
Indolalkylamine metabolic proc.
Diterpenoid biosynthetic proc.
Diterpenoid metabolic proc.
Indole-containing compound biosynthetic proc.
Indole-containing compound metabolic proc.
Cellular biogenic amine metabolic proc.
Aromatic amino acid family metabolic proc.
Terpenoid biosynthetic proc.
Terpenoid metabolic proc.
Secondary metabolite biosynthetic proc.
Isoprenoid biosynthetic proc.
Isoprenoid metabolic proc.
Secondary metabolic proc.
Defense response
0 20 40 60 80
Fold Enrichment
N. of Genes
5
10
15
-log10(FDR)
4
5
6
Salt Resistant up specific
Cold acclimation
Response to hydrogen peroxide
Response to reactive oxygen species
Response to cold
Response to temperature stimulus
Response to heat
Response to water deprivation
Response to abscisic acid
Response to alcohol
Response to acid chemical
Response to inorganic substance
Response to lipid
Response to oxygen-containing compound
Response to abiotic stimulus
Response to hormone
Response to endogenous stimulus
Response to organic substance
Response to chemical
Response to stress
0 25 50 75 100
Fold Enrichment
N. of Genes
4
8
12
16
-log10(FDR)
3
4
5
6
7
8
Salt Susceptible up
and Salt Resistant up
Tyrosine catabolic proc.
Reg. of response to water deprivation
Pos. reg. of response to water deprivation
Cold acclimation
Response to water deprivation
Response to acid chemical
Response to heat
Cellular response to heat
Cellular amino acid catabolic proc.
Response to temperature stimulus
Carboxylic acid catabolic proc.
Response to inorganic substance
Protein folding
Response to alcohol
Response to abiotic stimulus
Response to oxygen-containing compound
Response to chemical
Response to stress
0 20 40 60
Fold Enrichment
N. of Genes
10
20
30
-log10(FDR)
2
3
4
Salt Susceptible up specific

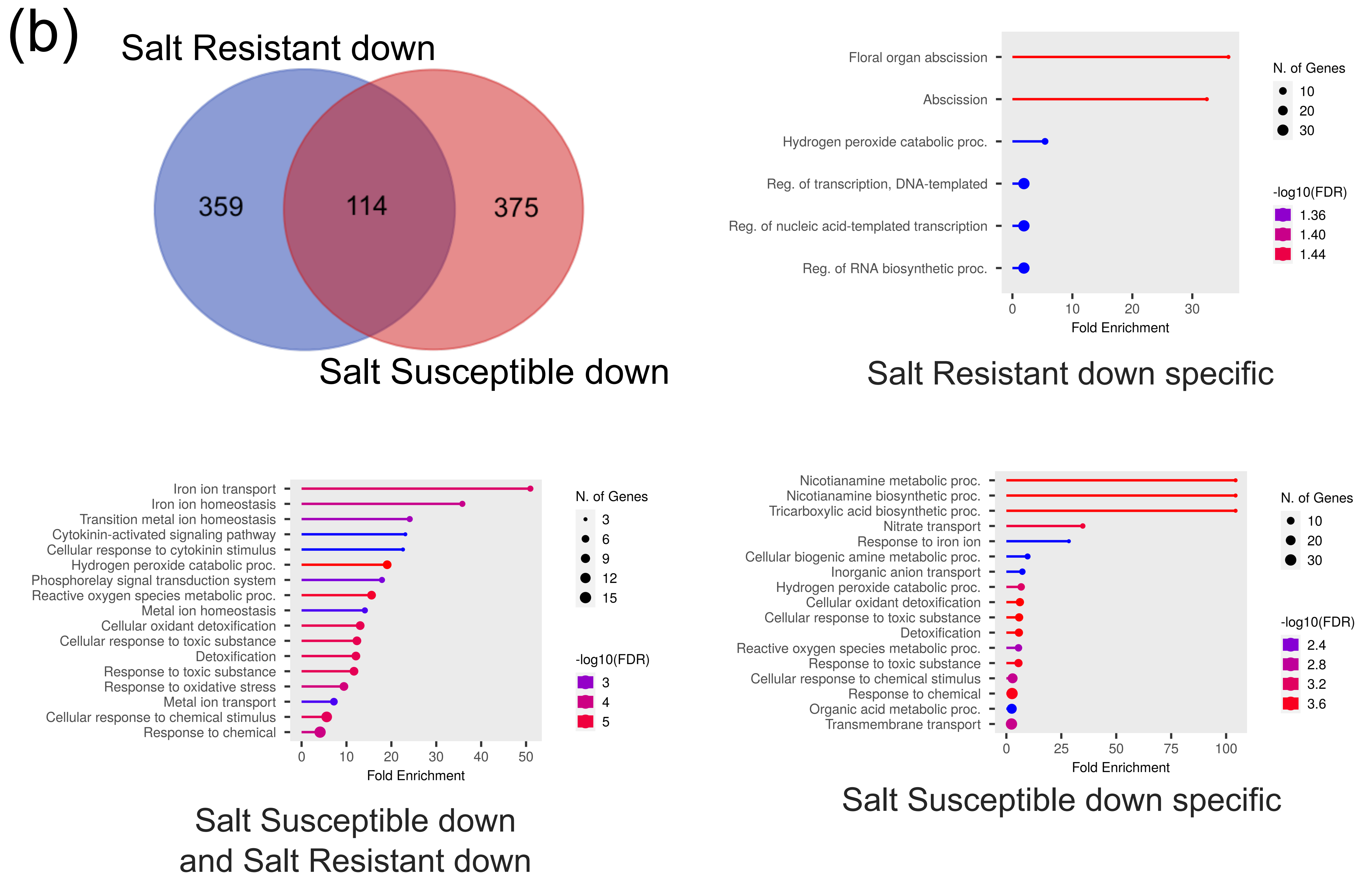

(b)
Salt Resistant down
359
114
375
Salt Susceptible down
Floral organ abscission
Abscission
Hydrogen peroxide catabolic proc.
Reg. of transcription, DNA-templated
Reg. of nucleic acid-templated transcription
Reg. of RNA biosynthetic proc.
0
10
20
30
Fold Enrichment
N. of Genes
10
20
30
-log10(FDR)
1.36
1.40
1.44
Salt Resistant down specific
Iron ion transport
Iron ion homeostasis
Transition metal ion homeostasis
Cytokinin-activated signaling pathway
Cellular response to cytokinin stimulus
Hydrogen peroxide catabolic proc.
Phosphorelay signal transduction system
Reactive oxygen species metabolic proc.
Metal ion homeostasis
Cellular oxidant detoxification
Cellular response to toxic substance
Detoxification
Response to toxic substance
Response to oxidative stress
Metal ion transport
Cellular response to chemical stimulus
Response to chemical
0
10
20
30
40
50
Fold Enrichment
N. of Genes
3
6
9
12
15
-log10(FDR)
3
4
5
Salt Susceptible down
and Salt Resistant down
Nicotianamine metabolic proc.
Nicotianamine biosynthetic proc.
Tricarboxylic acid biosynthetic proc.
Nitrate transport
Response to iron ion
Cellular biogenic amine metabolic proc.
Inorganic anion transport
Hydrogen peroxide catabolic proc.
Cellular oxidant detoxification
Cellular response to toxic substance
Detoxification
Reactive oxygen species metabolic proc.
Response to toxic substance
Cellular response to chemical stimulus
Response to chemical
Organic acid metabolic proc.
Transmembrane transport
0
25
50
75
100
Fold Enrichment
N. of Genes
10
20
30
-log10(FDR)
2.4
2.8
3.2
3.6
Salt Susceptible down specific

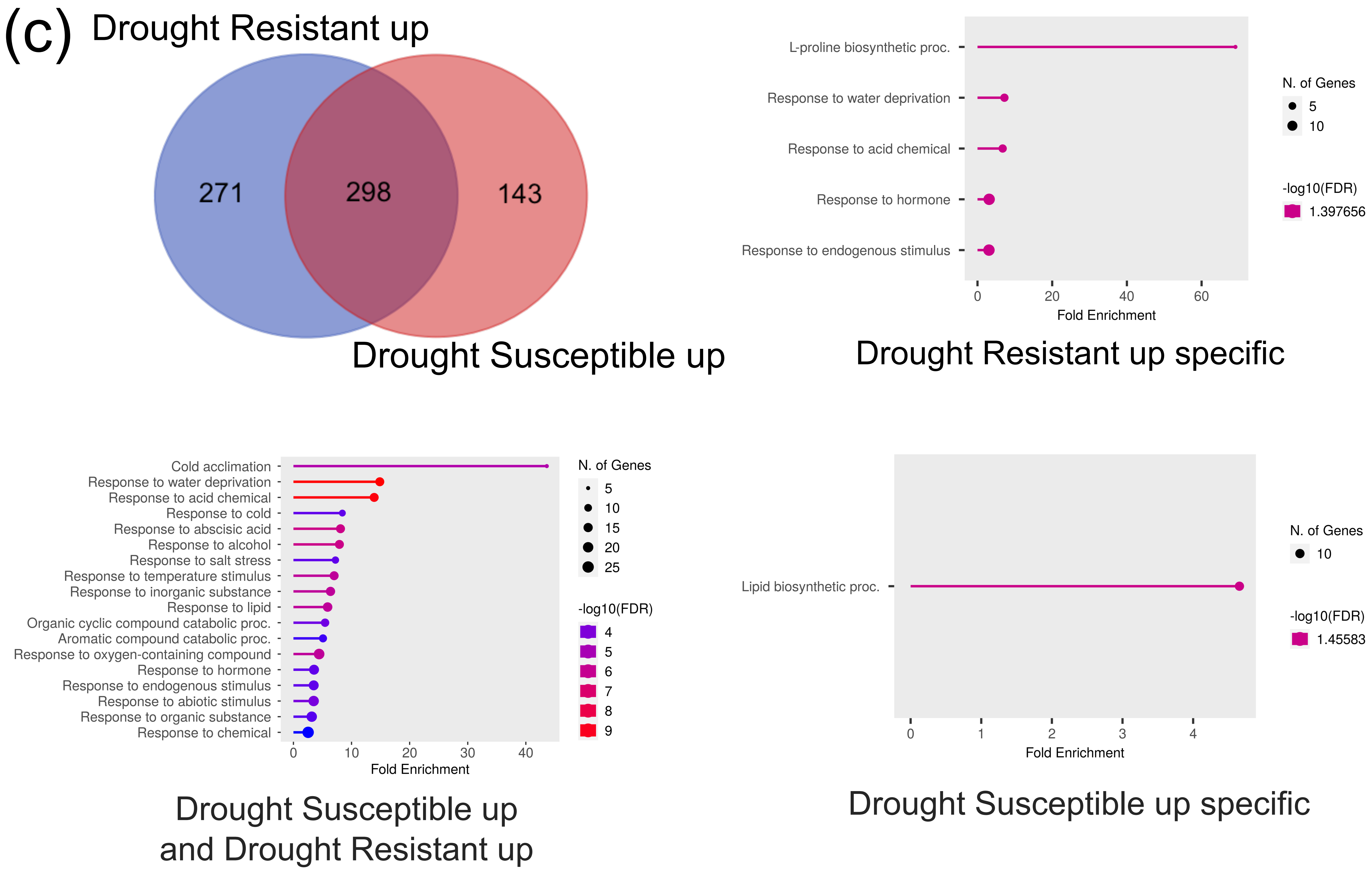
(c) Drought Resistant up
271
298
143
Drought Susceptible up
L-proline biosynthetic proc.
Response to water deprivation
Response to acid chemical
Response to hormone
Response to endogenous stimulus
0
20
40
60
Fold Enrichment
N. of Genes
5
10
-log10(FDR)
1.397656
Drought Resistant up specific
Cold acclimation
Response to water deprivation
Response to acid chemical
Response to cold
Response to abscisic acid
Response to alcohol
Response to salt stress
Response to temperature stimulus
Response to inorganic substance
Response to lipid
Organic cyclic compound catabolic proc.
Aromatic compound catabolic proc.
Response to oxygen-containing compound
Response to hormone
Response to endogenous stimulus
Response to abiotic stimulus
Response to organic substance
Response to chemical
0
10
20
30
40
Fold Enrichment
N. of Genes
5
10
15
20
25
-log10(FDR)
4
5
6
7
8
9
Drought Susceptible up
and Drought Resistant up
Lipid biosynthetic proc.
0
1
2
3
4
Fold Enrichment
N. of Genes
10
-log10(FDR)
1.45583
Drought Susceptible up specific

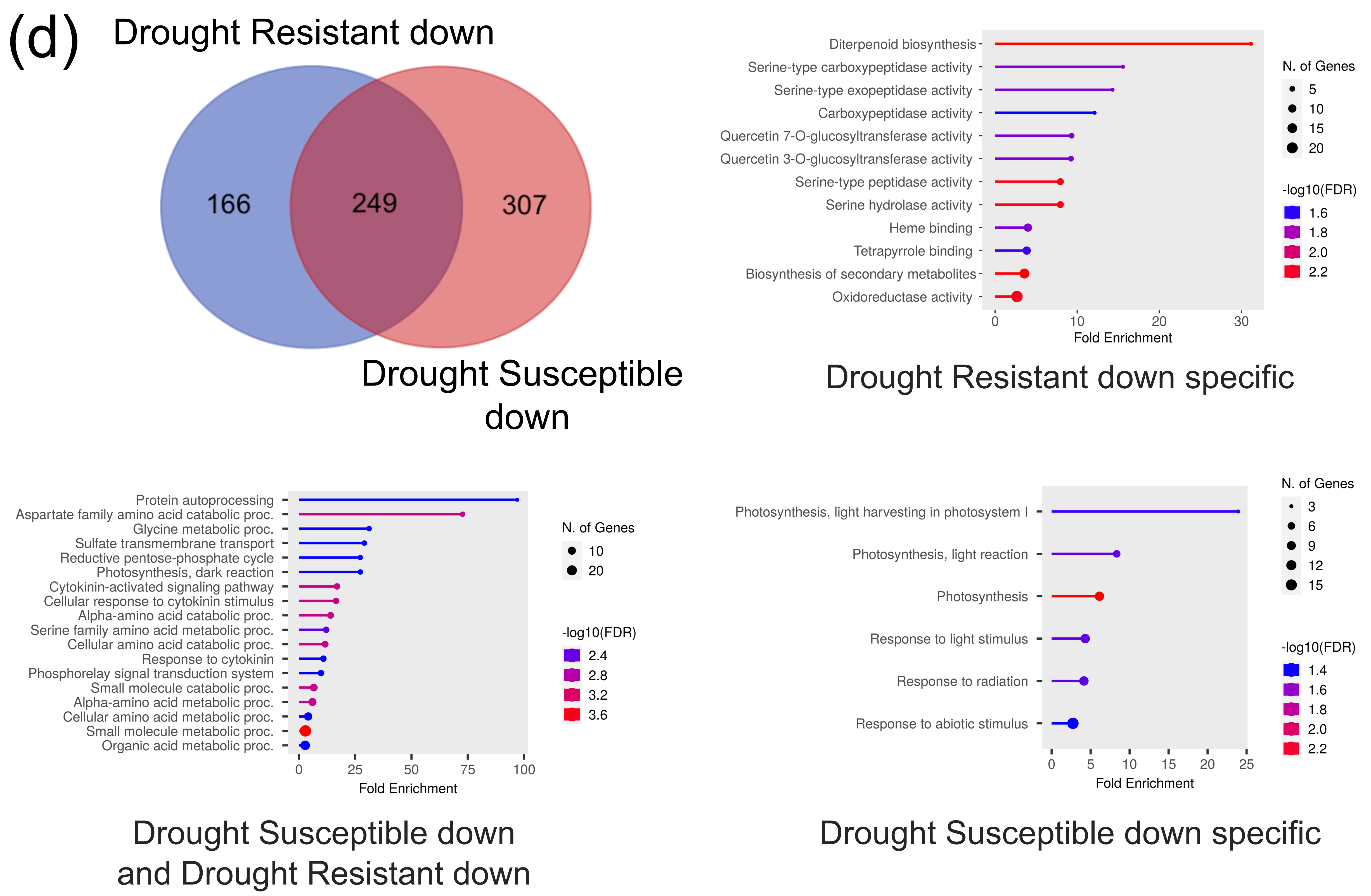
(d)
Drought Resistant down
166
249
307
Drought Susceptible down
Diterpenoid biosynthesis
Serine-type carboxypeptidase activity
Serine-type exopeptidase activity
Carboxypeptidase activity
Quercetin 7-O-glucosyltransferase activity
Quercetin 3-O-glucosyltransferase activity
Serine-type peptidase activity
Serine hydrolase activity
Heme binding
Tetrapyrrole binding
Biosynthesis of secondary metabolites
Oxidoreductase activity
0 10 20 30
Fold Enrichment
N. of Genes
5
10
15
20
-log10(FDR)
1.6
1.8
2.0
2.2
Drought Resistant down specific
Protein autoprocessing
Aspartate family amino acid catabolic proc.
Glycine metabolic proc.
Sulfate transmembrane transport
Reductive pentose-phosphate cycle
Photosynthesis, dark reaction
Cytokinin-activated signaling pathway
Cellular response to cytokinin stimulus
Alpha-amino acid catabolic proc.
Serine family amino acid metabolic proc.
Cellular amino acid catabolic proc.
Response to cytokinin
Phosphorelay signal transduction system
Small molecule catabolic proc.
Alpha-amino acid metabolic proc.
Cellular amino acid metabolic proc.
Small molecule metabolic proc.
Organic acid metabolic proc.
0 25 50 75 100
Fold Enrichment
N. of Genes
10
20
-log10(FDR)
2.4
2.8
3.2
3.6
Drought Susceptible down and Drought Resistant down
Photosynthesis, light harvesting in photosystem I
Photosynthesis, light reaction
Photosynthesis
Response to light stimulus
Response to radiation
Response to abiotic stimulus
0 5 10 15 20 25
Fold Enrichment
N. of Genes
3
6
9
12
15
-log10(FDR)
1.4
1.6
1.8
2.0
2.2
Drought Susceptible down specific
