# Supplementary material for: Meta-analysis of public RNA-sequencing data of drought and salt stresses in different phenotypes of resistant and susceptible Oryza sativa cultivars
Source: Quant Plant Biol. 2025 Sep 5;6:e27. doi: 10.1017/qpb.2025.10020 (PMC12451249; doi:10.1017/qpb.2025.10020)

(a)

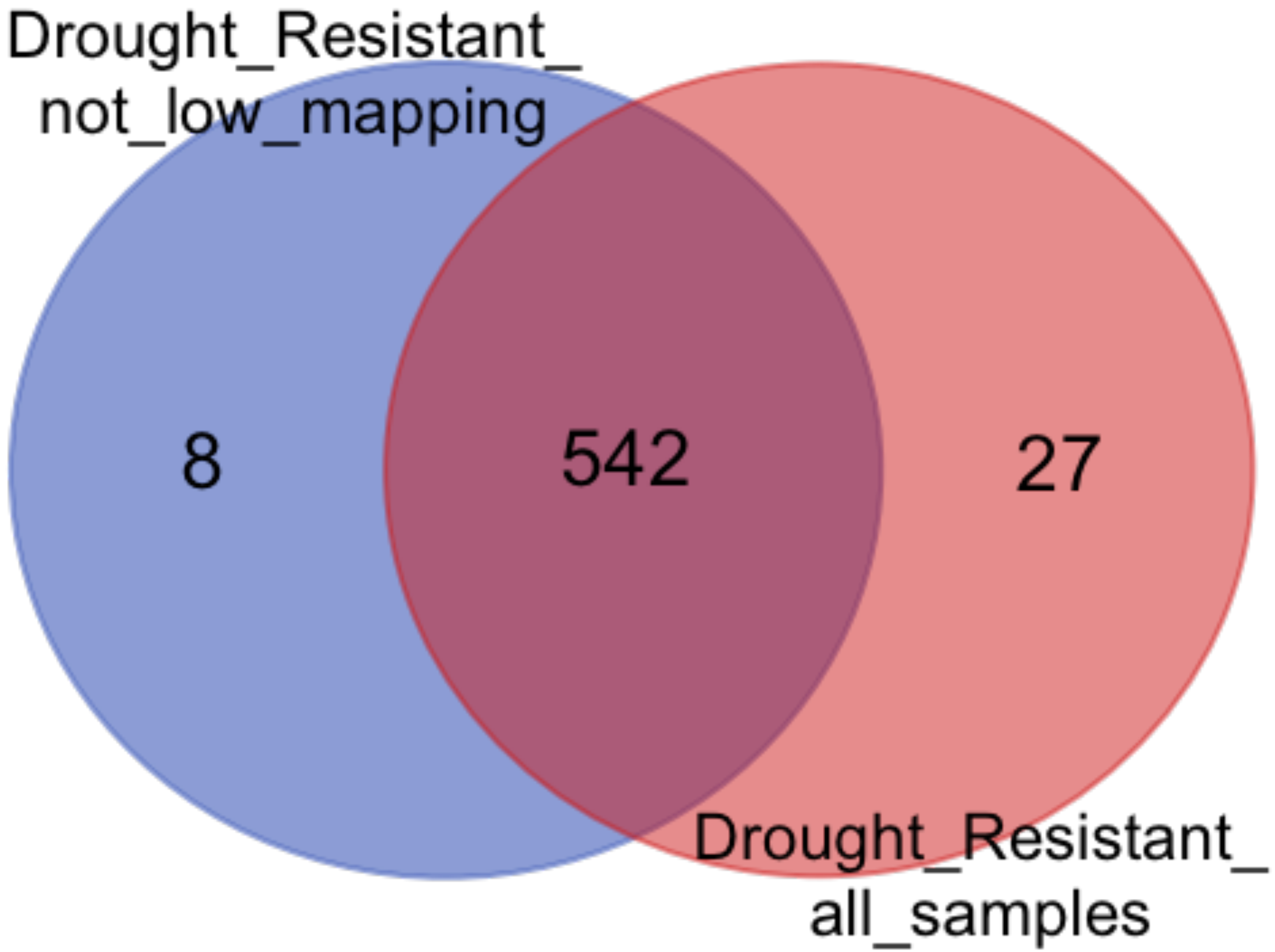
Drought_Resistant_
not_low_mapping
8
542
27
Drought_Resistant_
all_samples

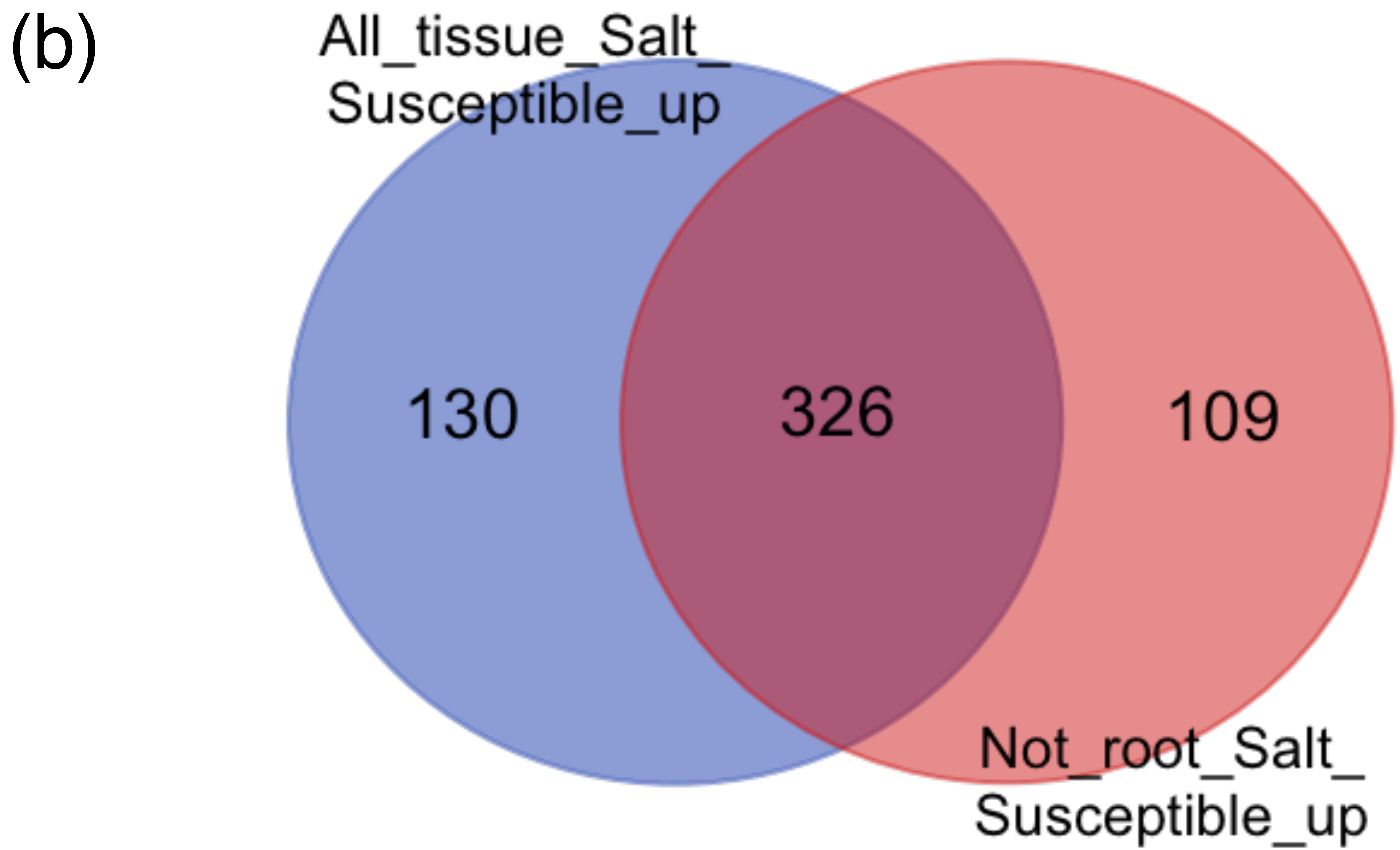
(b)
All_tissue_Salt_
Susceptible_up
130
326
109
Not_root_Salt_
Susceptible_up

(c) Not_root_Salt_Susceptible_up_and_All_tissue_Salt_Susceptible_up_326genes_overlap

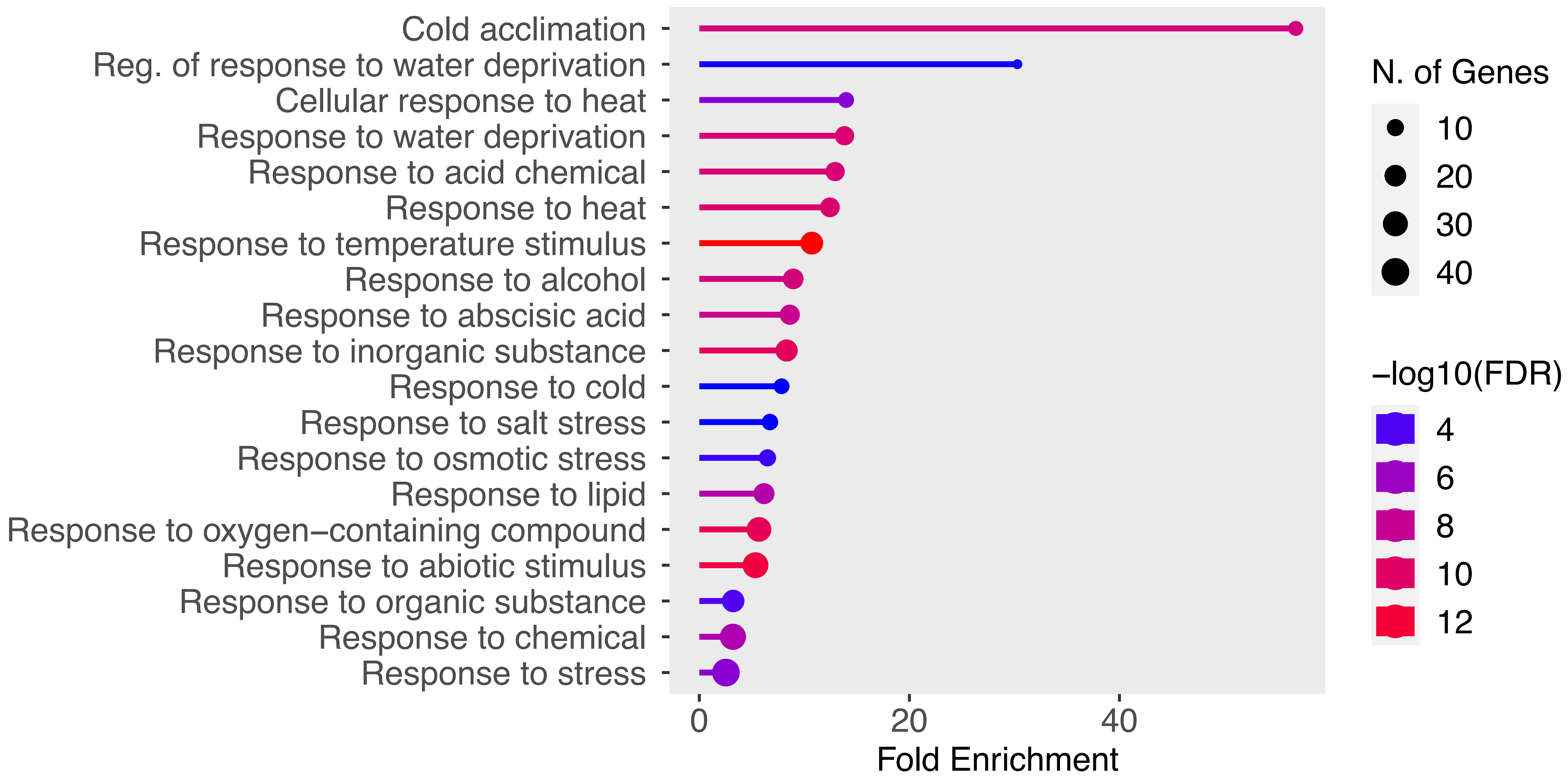

(d) All_tissue_Salt_Susceptible_up_456genes

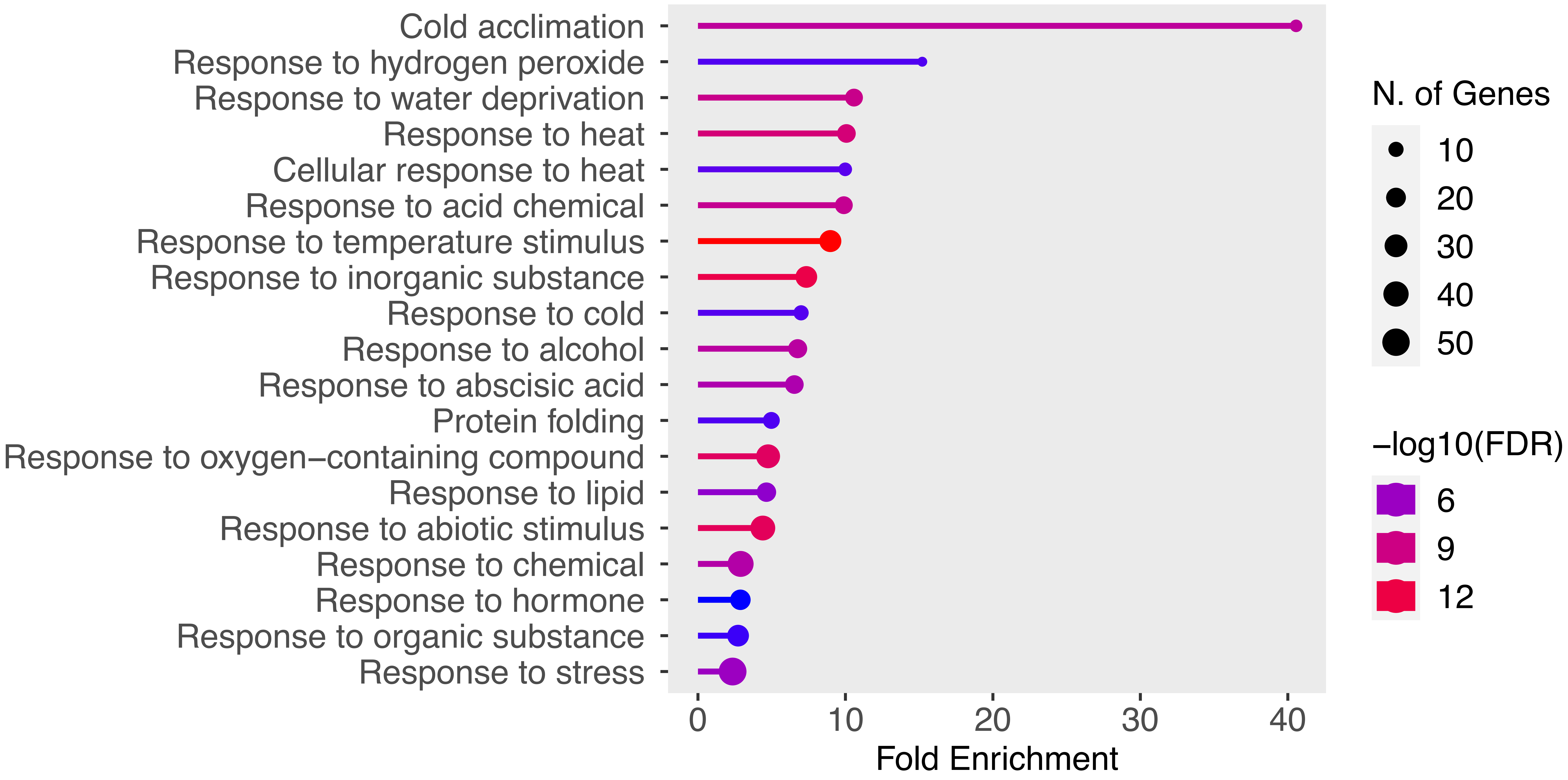

Supplement: Shintani and Bono supplementary material [file S2632882825100209sup001.zip › Supplementary_FigureS8.pdf]
