# Supplementary figures and images for: Meta-analysis of public RNA-sequencing data of drought and salt stresses in different phenotypes of resistant and susceptible Oryza sativa cultivars
Source: Quant Plant Biol. 2025 Sep 5;6:e27. doi: 10.1017/qpb.2025.10020 (PMC12451249; doi:10.1017/qpb.2025.10020)

Salt stress

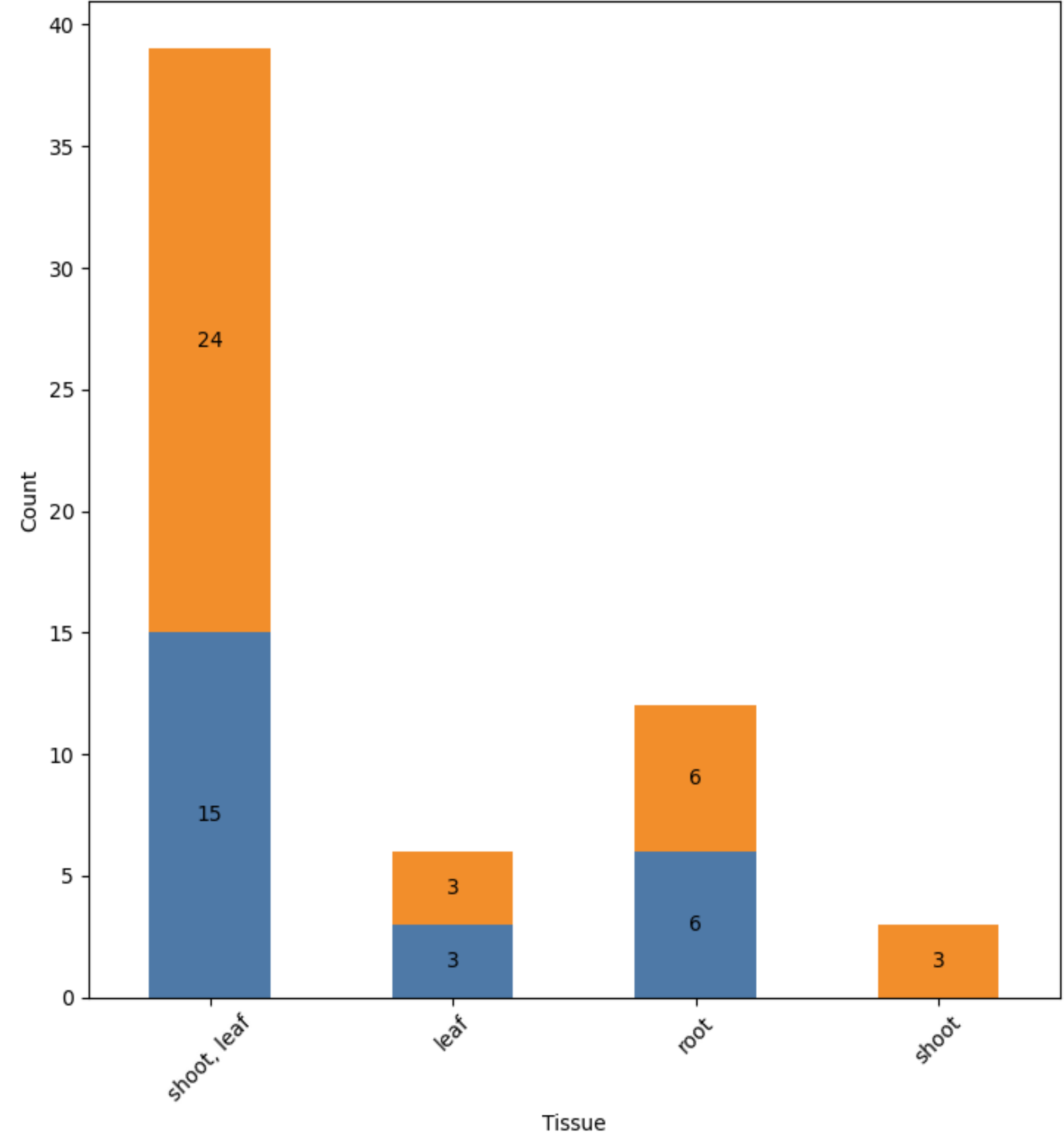

Drought stress

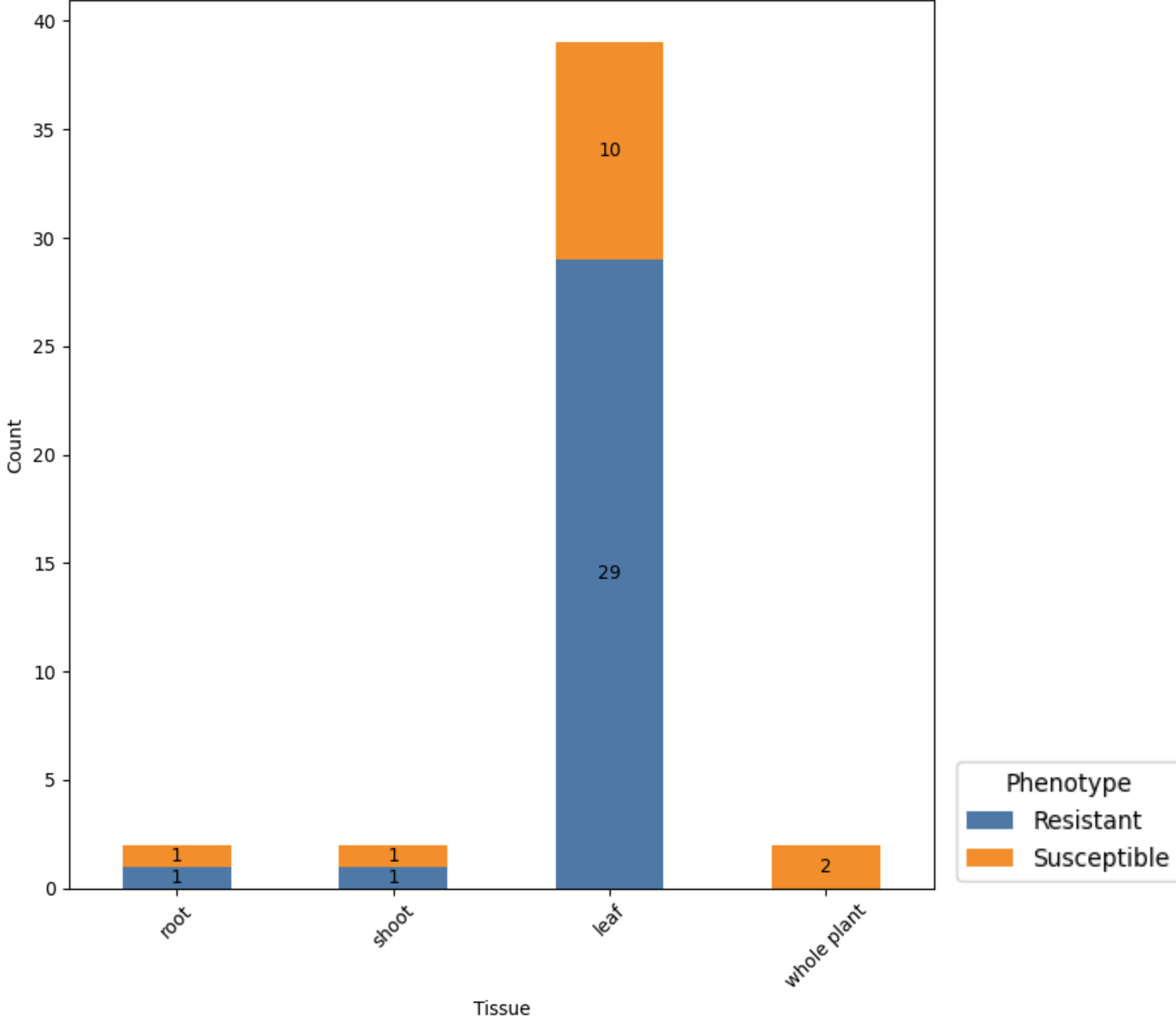

Supplement: Shintani and Bono supplementary material [file S2632882825100209sup001.zip › Supplementary_FigureS1.pdf]

(a)

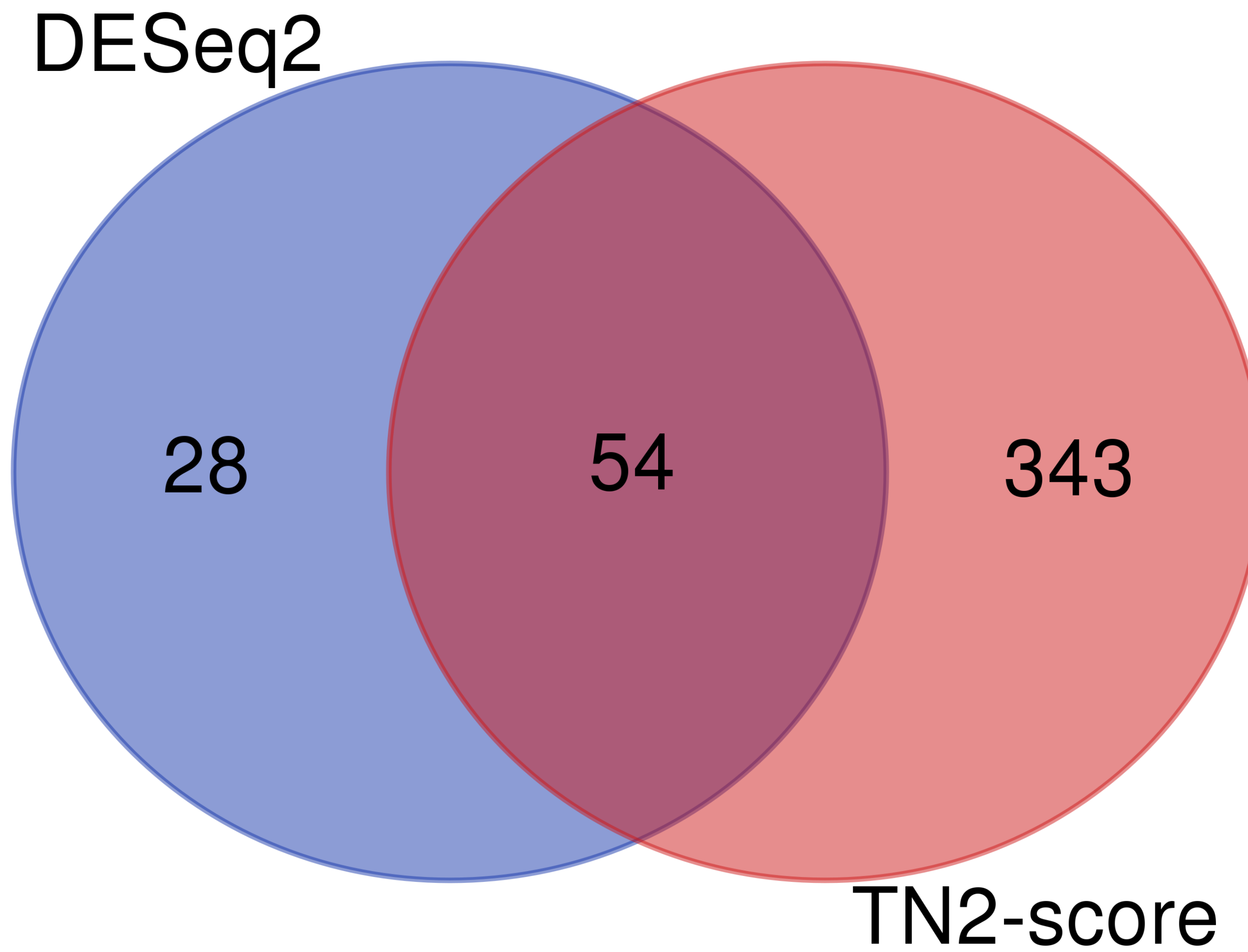

# (b) Overlap 54genes

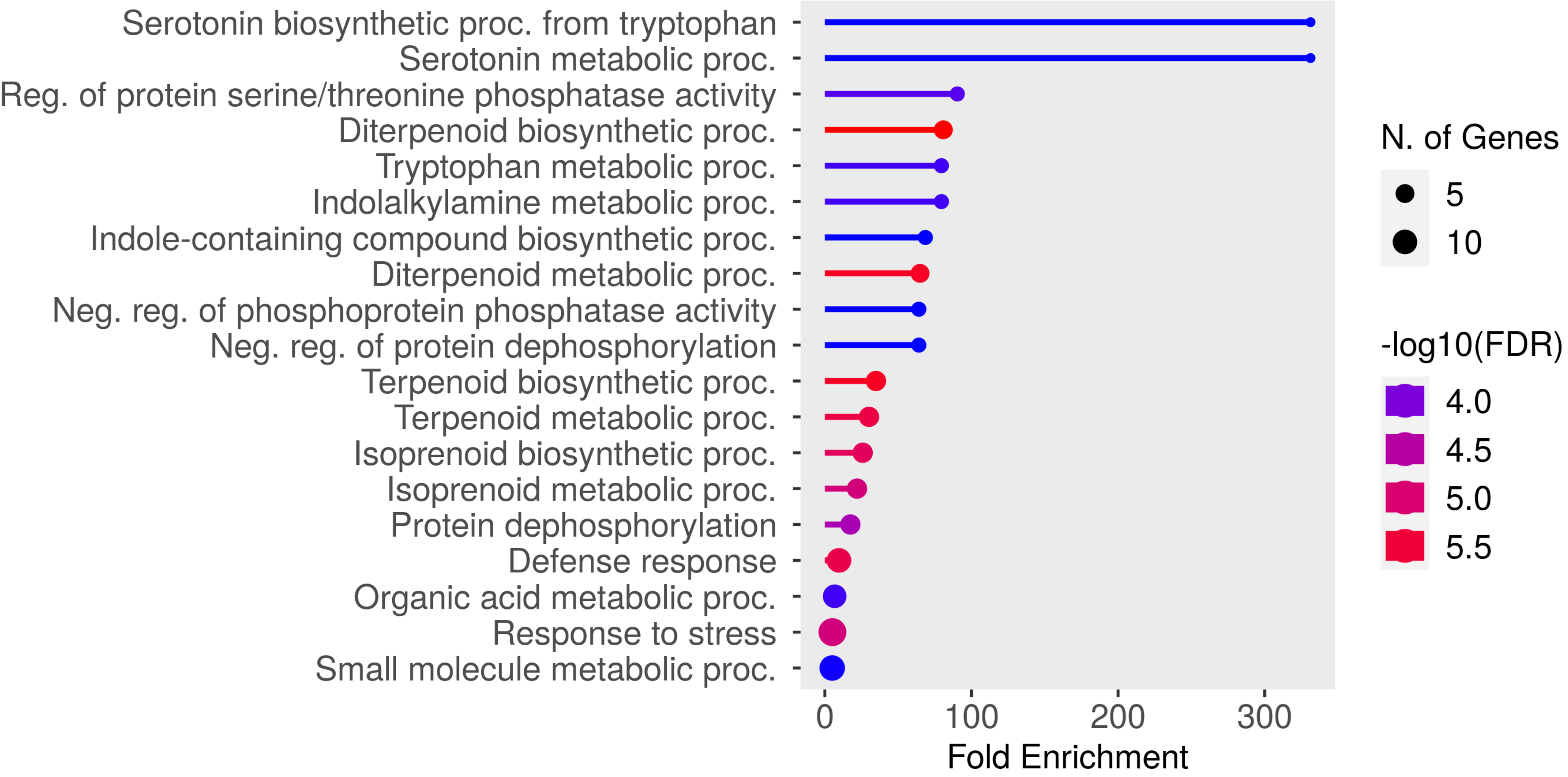

(c) DESeq2\_unique 28genes

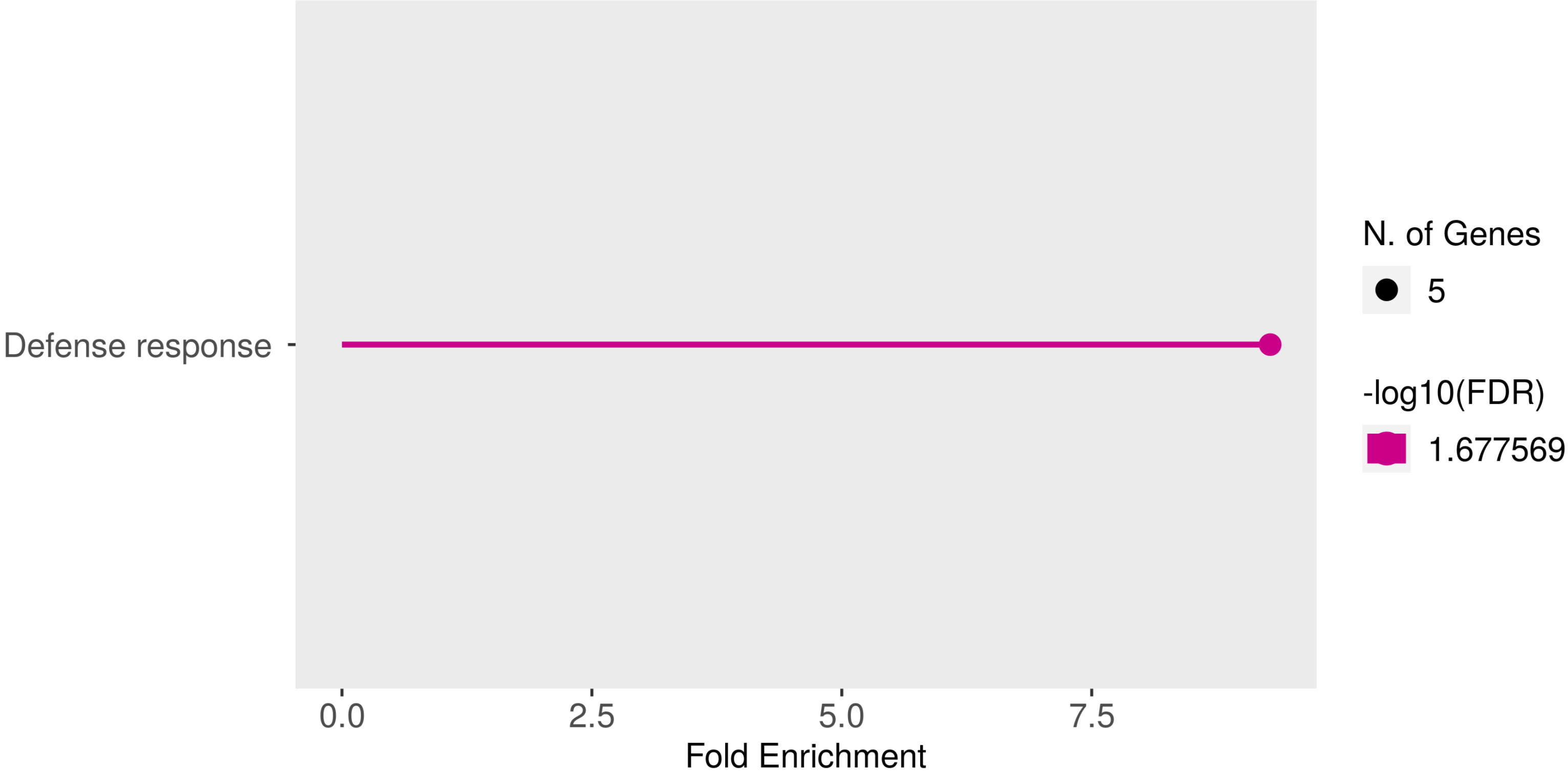

(d) TN2\_unique 343genes

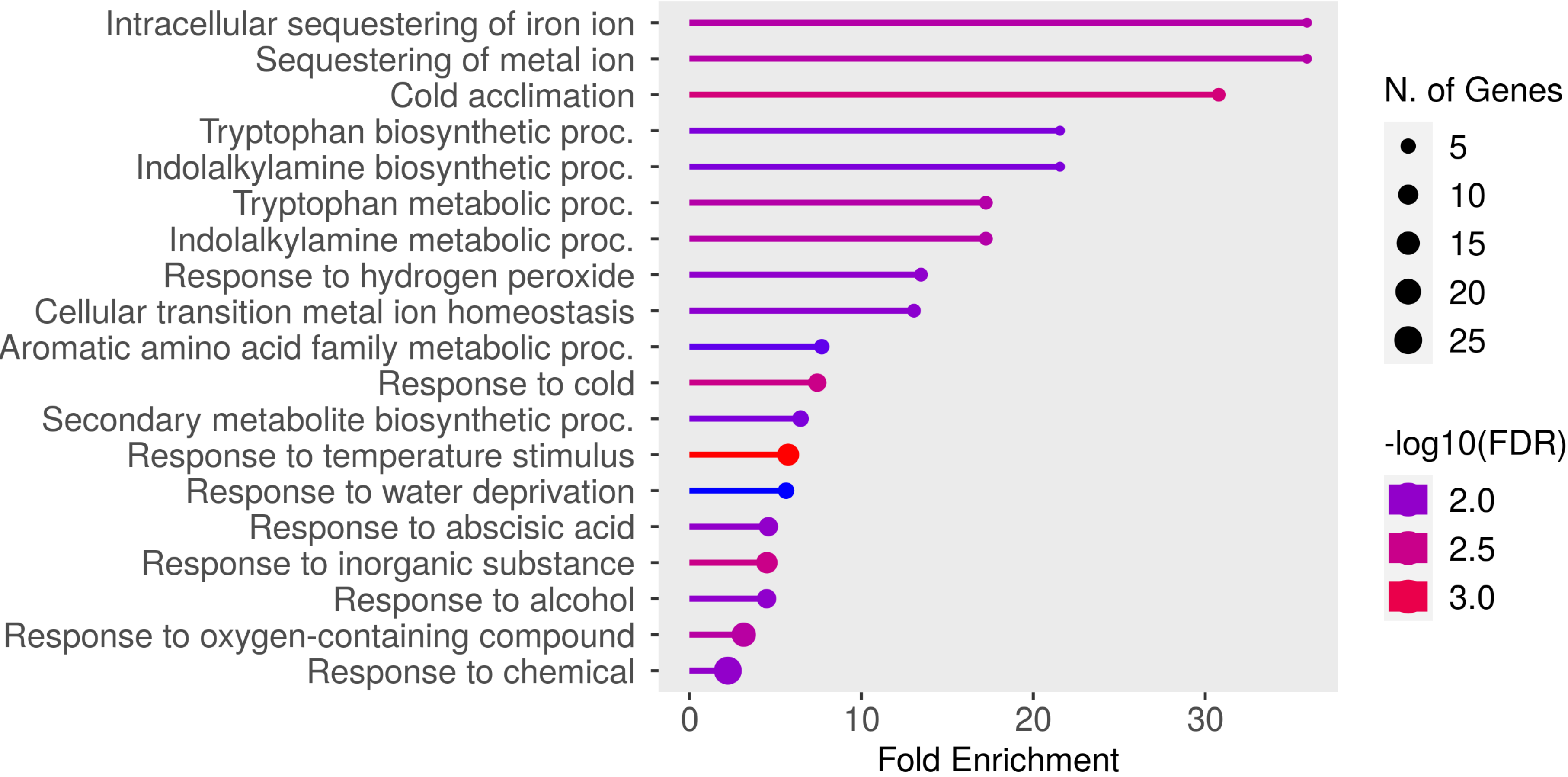

Supplement: Shintani and Bono supplementary material [file S2632882825100209sup001.zip › Supplementary_FigureS3.pdf]

(a)

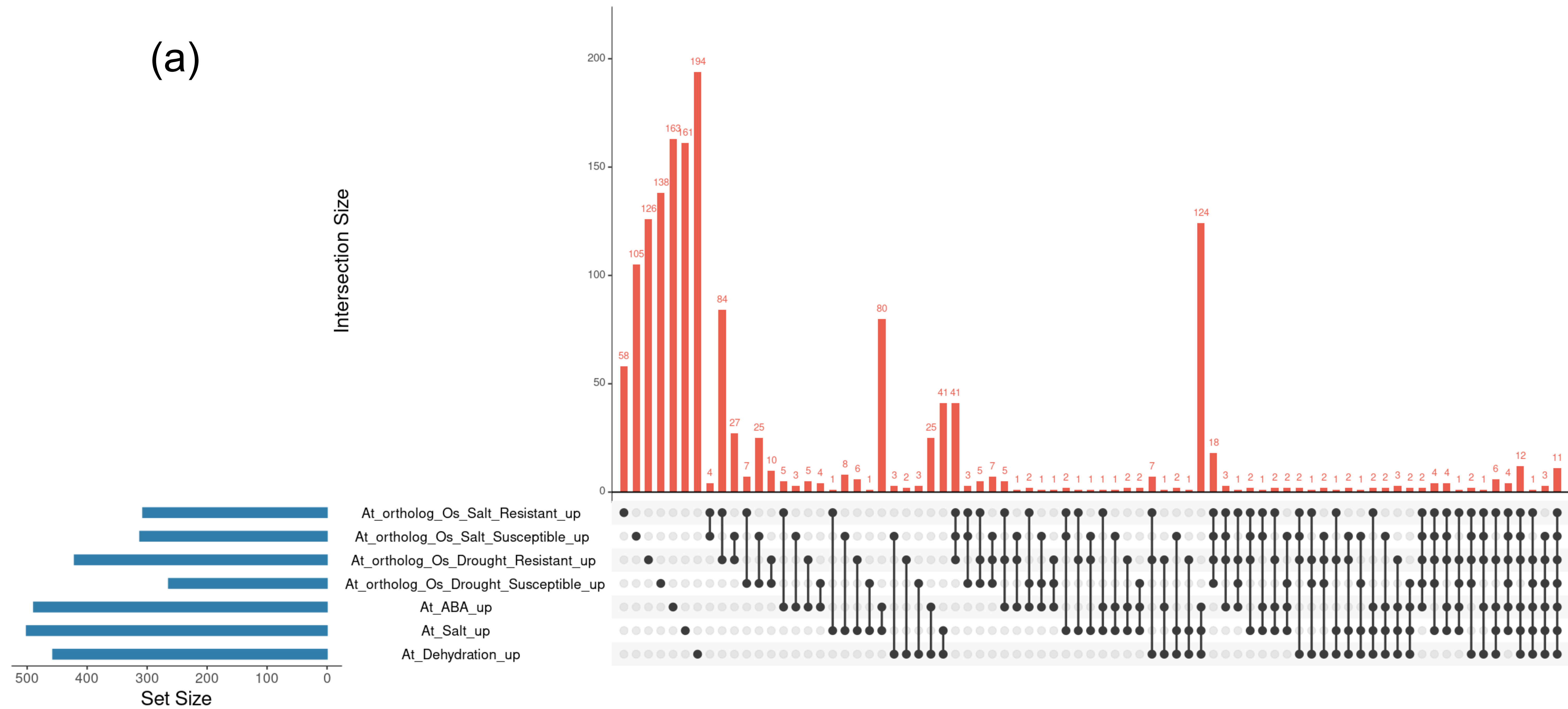

(b)

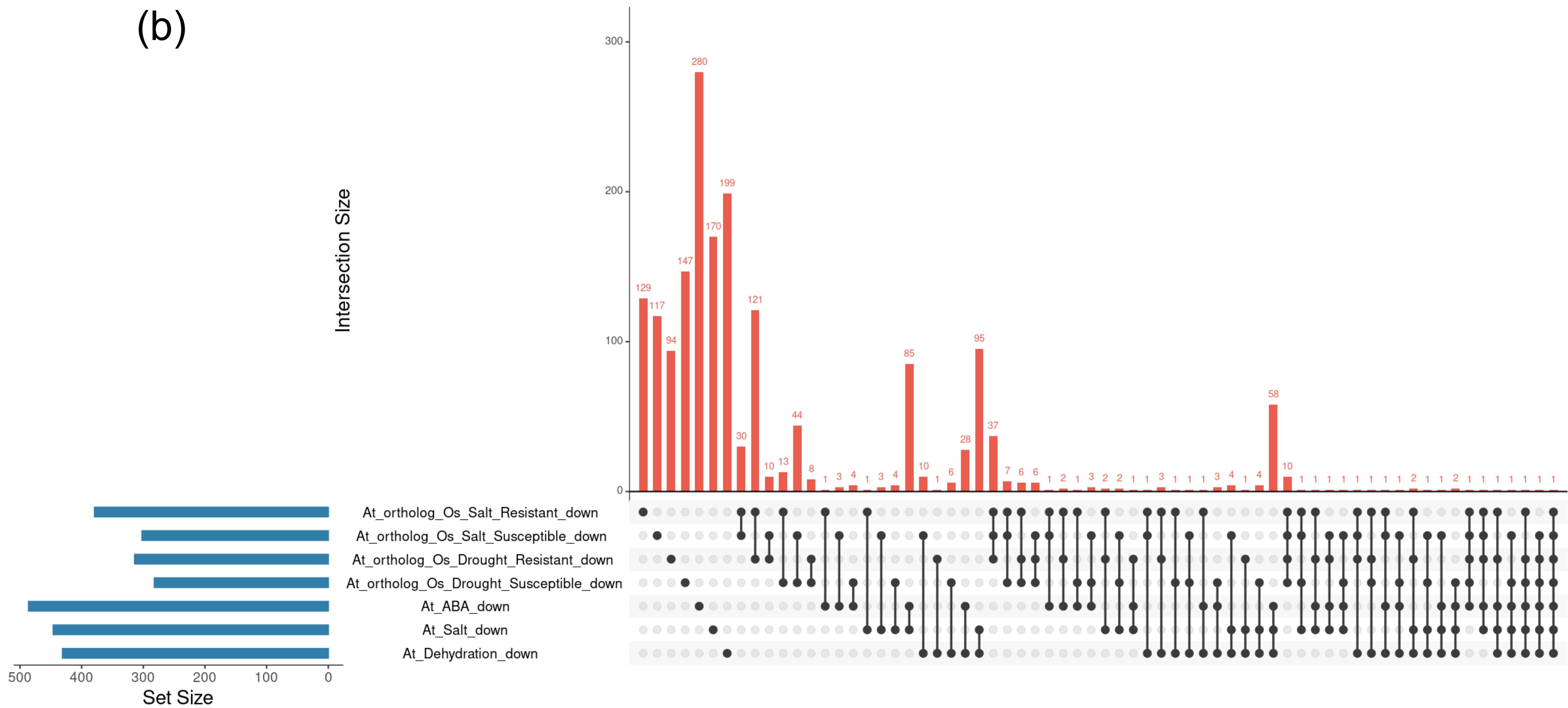

Supplement: Shintani and Bono supplementary material [file S2632882825100209sup001.zip › Supplementary_FigureS7.pdf]
